# Modeling Human Thyroid Development by Fetal Tissue‐Derived Organoid Culture

**DOI:** 10.1002/advs.202105568

**Published:** 2022-01-22

**Authors:** Jianqing Liang, Jun Qian, Li Yang, Xiaojun Chen, Xiaoning Wang, Xinhua Lin, Xiaoyue Wang, Bing Zhao

**Affiliations:** ^1^ State Key Laboratory of Genetic Engineering School of Life Sciences Human Phenome Institute Zhongshan Hospital Fudan University Shanghai 200438 China; ^2^ State Key Laboratory of Medical Molecular Biology Department of Biochemistry and Molecular Biology Institute of Basic Medical Sciences Chinese Academy of Medical Sciences School of Basic Medicine Peking Union Medical College Beijing 100730 China; ^3^ Obstetrics and Gynecology Hospital of Fudan University Shanghai Key Laboratory of Female Reproductive Endocrine Related Diseases Shanghai 200011 China; ^4^ School of Laboratory Medicine and Biotechnology Southern Medical University School of Biology and Biological Engineering South China University of Technology Guangzhou 510000 China

**Keywords:** cAMP signaling, cell atlas, fetal thyroid organoids, human thyroid development, in vitro maturation

## Abstract

Euthyroidism is of profound importance for lifetime health. However, the early diagnosis or therapeutics of thyroid developmental defects has not been established, mainly due to limited understanding of human thyroid development and a lack of recapitulating research model. Herein, the authors elaborate the cell atlas and potential regulatory signaling of the evolution of heterogeneous thyrocyte population from 12 to 16 gestational weeks. Moreover, they establish a long‐term culture of human fetal thyroid organoids (hFTOs) system, which retains the fetal thyroid lineages and molecular signatures, as well as the ability to generate functional human thyroid follicles post mice renal transplantation. Notably, cAMP signaling activation in hFTOs by forskolin boosts the maturation of follicle and thus thyroid hormone T4 secretion, which recapitulates the key developmental events of fetal thyroid. Employing this ex vivo system, it is found that enhanced chromatin accessibility at thyroid maturation genes (such as *TPO* and *TG*) loci permits the transcription for hormone production. This study provides the cell atlas of and an organoid model for human thyroid development, which will facilitate thyroid research and prospective medicine.

## Graphical abstract

The study elaborates the cell atlas and potential regulatory signaling of the evolution of heterogeneous thyrocyte population from the human 12–16 gestational weeks fetuses. A human fetal thyroid organoids system recapitulates fetal thyroid maturation process that cAMP signaling activation in organoids by forskolin boosts the maturation of follicle and the secretion of thyroid hormone T4.

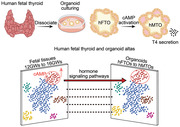

## 1 Introduction

Normal thyroid function has profound impacts on lifelong health. Both the deficiency and excess of thyroid hormones were shown to be associated with potentially devastating health consequences,^[^ ^1^ ^]^ particularly in pregnancy and childhood.^[^ ^2^ ^]^ Despite its vital functions, few studies have focused on human thyroid development. This is mainly because the developing embryo utilizes the mother's thyroid hormone to ensure the central nervous system development; cases where the mother has insufficient thyroid hormones during pregnancy, thyroid hormone supplements can usually solve the issue.^[^ ^3^ ^]^

However, increasing occurrences of adolescent thyroid disorders^[^ ^4^ ^]^ and limited diagnosis before severe manifestations greatly challenged the absence of knowledge of human thyroid development.^[^ ^5^ ^]^ As key developmental events have not been established at the cellular or molecular level, it is rather difficult to determine whether adolescent thyroid disorders originate from embryonic developmental defects. Besides, whether thyroxine supplement taken by pregnant women is safe for fetal thyroid also needs to be carefully evaluated. In addition, most congenital hypothyroidism is caused by thyroid dysgenesis affecting nearly 1 in every 2000 newborns, yet its underlying causes are poorly understood.^[^ ^6^ ^]^

Human thyroid development follows a precisely time‐based gene expression program^[^ ^7^ ^]^ and its terminal differentiation can be divided into colloid formation stage (during 7–11 gestational weeks [GWs]) and follicular growth stage (starts from 12GWs).^[^ ^8^ ^]^ To date, mouse genetics have identified several factors that determine thyroid specification, morphogenesis, and terminal differentiation.^[^ ^7^, ^9^ ^]^ In contrast, human studies only reported the expression pattern of specific genes in samples,^[^ ^10^ ^]^ or the transcriptome of fetal thyroid at premature stage (7–11GWs).^[^ ^11^ ^]^ The knowledge on the initiation of human fetal thyroid functional maturation at follicular growth stage is still limited. Recent single‐cell RNA sequencing (scRNA‐seq) of adolescent and adult zebrafish thyroid showed heterogeneity in developmental thyroid,^[^ ^12^ ^]^ highlighting the potential of single‐cell molecular characterization in understanding thyroid maturation. Even so, the cellular composition dynamics of fetal thyroid maturation in mammal, especially in human, remain unclear. Hence, a genome‐wide transcriptome view of human thyroid development, especially in single‐cell resolution, is urgently needed for the understanding of human thyroid development and the discovery of potential early diagnosis markers and therapeutics for thyroid diseases.

An organoid model system to recapitulate human thyroid development is also required for the comprehensive functional research of human thyroid development. Recently, studies have reported that ESC‐ or iPSC‐derived and adult tissue‐derived thyroid organoids exhibit principal thyroid characteristics and potential transplantation feasibility.^[^ ^13^ ^]^ However, due to the lack of time‐equivalent information of human fetal thyroid tissue or organoids as a reference, the in vivo developmental events and cell atlas in specific time windows are still unclear, which also limits the use of PSC‐based systems in modeling thyroid development. Therefore, it is important to generate human fetal thyroid organoids (hFTOs) from fetal tissues and establish an ex vivo maturation system. Understanding how human fetal thyrocytes achieve differentiation and consequent hormone production would improve strategies for directed differentiation of pluripotent cells.

In this study, we interrogated the transcriptome of human fetal thyroid at single‐cell resolution, capturing a mature state subpopulation of thyrocytes and exploring insight into the mechanism of human thyroid development. We further established a long‐term hFTOs culture system using fetal primary thyroid tissue. The hFTOs not only preserved the lineage features and molecular characteristics of human fetal thyroid tissue, but also retained the ability to generate human thyroid follicles post mice renal transplantation. Importantly, we induced hFTOs maturation by activating cAMP signaling and obtained hormone‐secreting human thyroid organoids in vitro, which well mimicked the key thyroid developmental events. Using RNA sequencing (RNA‐seq) and ATAC‐seq, we explored the transcriptional changes and chromatin accessibility in fetal thyroid organoids, confirming the similarity of hFTO maturation by cAMP signal to the development of fetal follicular cells.

## 2 Results

### 2.1 Mapping the Gene Expression Landscape of Cells in Human Fetal Thyroid Gland

To understand the cellular composition and heterogeneity of developing human fetal thyroid gland, we used a 10× genomics‐based platform to profile the transcriptome, at the single‐cell level, of the entire human fetal thyroid glands from two different GWs: 12GWs, when the thyroid follicles undergo progressive growth and are ready for the onset of hormone synthesis;^[^ ^8^ ^]^ and 16GWs, when the thyroid gland approaches structural maturity^[^ ^14^ ^]^ (Figure S1, Supporting Information). All the samples were obtained with the informed consent by the patients who had made legally elective abortion. The fetal thyroid gland was excised from the donor fetus and the connective tissues were cleaned up under a stereomicroscope, followed by minced and digested process (Experimental Section). Around 8000 single cells from each sample of 12GWs, 16GWs_1 and 16GWs_2 were then input into the 10× genomics protocol. After quality control, we captured 18 534 cells with a median of 2784 genes expressed per cell, providing a high‐resolution atlas of human fetal thyroid glands (Figure S2A, Supporting Information).

**Figure 1:**
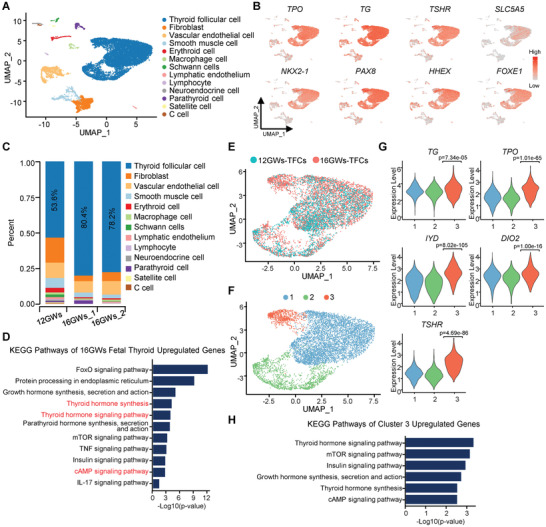
Single‐cell transcriptome survey of developing human fetal thyroid gland. A) UMAP of fetal thyroid tissue from two different gestational weeks (12GWs and 16GWs), with cells colored by cell type. B) UMAP showing the expression levels of thyroid follicular cell markers and four key transcriptional factors. C) The proportion of diverse cell types in human thyroid gland at two different developmental time points (12GWs and 16GWs). D) KEGG analysis of upregulated genes that are differentially expressed in thyroid follicular cells of 16GWs, compared to 12GWs. E) UMAP of thyroid follicular cells (TFCs) from 12GWs (blue dots) and 16GWs (red dots) of fetuses. F) UMAP plot showing three cell clusters of TFCs partitioned by single‐cell transcriptome. G) Violin plot showing marker genes expression of the three cell clusters in TFCs. *p*‐value was performed by Wilcoxon Rank Sum test between cluster 3 against other clusters. H) KEGG analysis of genes differentially expressed in cluster3 of TFCs in (F).

As visualized using uniform manifold approximation and projection (UMAP)^[^ ^15^ ^]^ (**Figure** **1**A), our atlas comprises all the main known thyroid gland cell types defined by the expression of canonical marker genes (Figure S2B,C, Supporting Information), including *EPCAM+* epithelial cells, fibroblasts, endothelial cells, muscle cells, immune cells, and neuron cells. Based on the expression of thyroid marker genes (*TPO*, *TG*, *TSHR*, and *SLC5A5*) (Figure 1B), we identified the largest group of *EPCAM+* epithelial cells as thyroid follicular cells (TFCs), which are responsible for the synthesis and secretion of thyroid hormones. As expected, the four key thyroid transcriptional factors (*NKX2‐1*, *HHEX*, *PAX8*, and *FOXE1*) were highly expressed in TFCs as well (Figure 1B). In the *EPCAM*+ epithelial cells, we also identified other cell components of thyroid parenchyma, such as parathyroid cells (*PTH*+/*GCM2*+)^[^ ^16^ ^]^ (Figure 1A and Figure S2D,E, Supporting Information) and C cells (*CALCA*+)^[^ ^17^ ^]^ (Figure 1A and Figure S2F, Supporting Information). Because the clustering‐based cell annotation using whole transcriptome data inevitably has some inconsistencies with marker‐based cell annotation,^[^ ^18^ ^]^ low expression of *PAX8*/*NKX2‐1* in other cell types were not taken account (Figure S2G, Supporting Information). Cell component proportion analysis elucidated variations in cell composition during development. TFCs markedly increased in 16GWs (80.4% and 78.2%), compared to 12GWs (53.6%) (Figure 1C), which is consistent with previous reports that the rapid proliferation of follicular epithelial cells synchronizes with the initiation of hormone synthesis beginning around 12 GWs.^[^ ^8^ ^]^ The consistency of two cases of 16GWs also reflects the reproducibility and fidelity of our data (Figure 1C and Figure S2A, Supporting Information).

### 2.2 A Subpopulation of Follicular Cells Exhibited Hormone Secretion Function from 12 to 16 GWs

To better understand how thyroid develops at different fetal stages, we extracted all TFCs from thyroids at these two representative stages for detailed analysis. As expected, functional analysis revealed that upregulated genes in TFCs at 16GWs are enriched in the thyroid hormone synthesis pathway (Figure 1D). Moreover, other KEGG annotated pathways, such as IL‐17, TNF, FoxO, cAMP, and mTOR signaling pathway, are significantly associated with 16GWs upregulated genes, suggesting their roles in thyroid development between 12 to 16 GWs (Figure 1D). However, whether all or partial TFCs conducted hormone synthesis is not known. To understand the heterogeneity of TFCs, we reclustered the TFCs from the whole dataset into three subgroups (Figure 1E,F and Figure S2H, Supporting Information). Intriguingly, cluster 3 was strongly enriched in the 16GWs samples and was almost absent in 12GWs. Cluster 3 had higher expression of thyroid hormone synthesis‐related genes (*TG, TPO, DIO2, IYD*, and *TSHR*) than other TFCs clusters (Figure 1G). Pathway enrichment analysis of upregulated genes in cluster 3 identified 21 significantly enriched pathways from over 200 KEGG pathways (adjusted *p*‐value < 0.05, Table S3, Supporting Information), including “Growth hormone synthesis, secretion, and action” and “Thyroid hormone signaling pathway” (Figure 1H). These results indicate that cells in cluster 3 are at a more mature state that is characterized by active hormone synthesis and secretion.

### 2.3 Cell Fate Transition and Regulons in Human Thyroid Development

**Figure 2:**
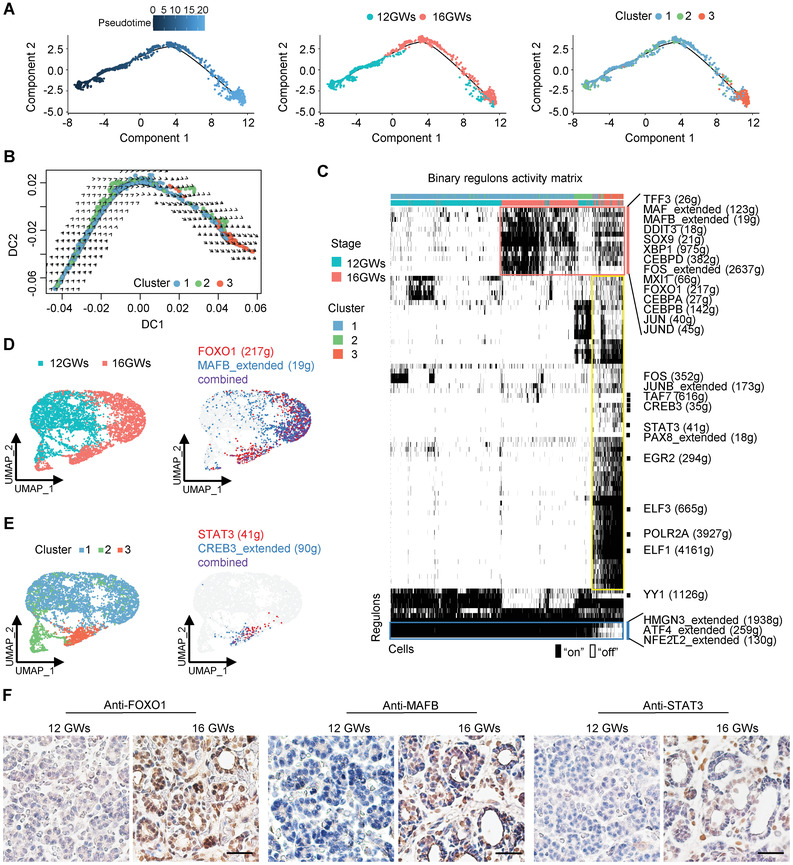
Cell fate transition and regulons during human thyroid development from 12 to 16 GWs. A) Left panel: Ordering of TFCs along a pseudotemporal trajectory using Monocle. Cells are colored by pseudotime. Middle panel: Position of TFCs from 12GWs (blue dots) and 16GWs (red dots) along the pseudotemporal trajectory. Right panel: Position of TFCs in different clusters along the pseudotemporal trajectory. B) Diffusion map showing RNA velocity for TFCs. For each cell, arrows indicate the location of the future cell state. The cells are colored by cluster identity as in the right panel of (A). C) Heatmap of regulon activity analyzed by SCENIC with default thresholds for binarization. The top two rows represent the stage and cluster state for each cell. “On” indicates active regulons; “Off” indicates inactive regulons. The representative regulons are listed on the right and the number of genes enriched in regulons is indicated in parenthesis. D) UMAP projection of the selected cells was labeled with sample stage (left) and the average binary regulon activity of representative genes FOXO1 and MAFB (right). E) UMAP projection of the selected cells were labeled with cluster stage (left) and the average binary regulon activity of STAT3 and CREB3 that are active specifically in cluster 3 (right). F) Immunohistochemical staining of FOXO1, MAFB, and STAT3 in thyroid tissues of 12GWs and 16GWs, and nuclei stain with hematoxylin. Scale bar = 100 µm.

To investigate cellular transitions of TFCs in this specific time window, we performed pseudotemporal ordering of TFCs from two timepoints using Monocle2. On the pseudotime plot, the cells from 12 GWs and 16 GWs distributed distinctly along the trajectory (**Figure** **2**A). Cluster 3, which was identified as the more mature TFC cells, was placed on the rightmost of the trajectory (Figure 2A). RNA velocity analysis was performed to confirm that the direction of differentiation is from cluster 1/2 to cluster 3 (Figure 2B). Clustering differentially expressed genes (DEGs) in TFCs between 12GWs and 16GWs, based on their pseudotemporal expression pattern, revealed different gene sets (Figure S3A–C, Supporting Information). Although some cells from 12GWs were included in cluster 3, KEGG analysis on cluster 3 cells from 12GWs exhibited that the hormone signaling pathway was not significantly enriched as those cells from 16GWs (Figure S3D,E, Supporting Information). These results suggest a distinction between 12GWs and 16GWs cells in cluster 3. It is possible that these cells play a role in initiating thyroid maturation. Together, our results demonstrate cellular heterogeneity in the thyroid gland and reveal the existence of one group of more mature follicular cells that initiate hormone synthesis function during fetal development.

To assess which transcription factors contribute to the differences in the expression of developing TFCs, we investigated the TF regulons activity using SCENIC. We found that activation of TF regulatory modules displayed a specific spatiotemporal pattern (Figure 2C). For example, a group of TFs (*TFF3, MAF, MAFB, DDIT3, SOX9, XBP1, CEBPD, FOS, MXI1, FOXO1, CEBPA, CEBPB, JUN*, and *JUND*) were activated restrictively in the cells of 16GWs (Figure 2C,D), indicating their roles in thyroid developmental regulation. Among these, *FOXO1* has been reported to be highly expressed in differentiated thyroid cells and is responsive to thyroid stimulating hormone (TSH);^[^ ^19^ ^]^ and *MAFB* regulates parathyroid development and hormone expression.^[^ ^20^ ^]^ Moreover, a large group of TFs were specifically active in cluster 3, including genes associated with the cAMP pathway, such as *FOS, JUNB, CREB3*, and *STAT3* (Figure 2C,E), while *HMGN3, ATF4*, and *NFE2L2* were turned off in cluster 3 TFCs (Figure 2C). Importantly, immunohistochemical staining showed that the FOXO1, MAFB, and STAT3 were highly expressed in the more mature 16GWs thyroid tissue but not in the 12GWs thyroid tissue (Figure 2F), suggesting indicated regulons may play important roles in fetal thyroid maturation. In addition, considering cAMP is a general second messenger which may be connected to G‐protein coupled 7TM receptors (GPCRs), we screened genes upregulated in 16GWs in comparison to 12GWs, and found several GPCRs, including *GPBP1*, *GPRC5A*, *ADGRV1*, *ADGRA3*, and *GPR183*, indicating these genes may contribute to the transduction of extracellular stimuli during thyroid developmental process (Figure S3F, Supporting Information).

### 2.4 Establishment of Long‐Term Human Fetal Thyroid Organoid Culture

**Figure 3:**
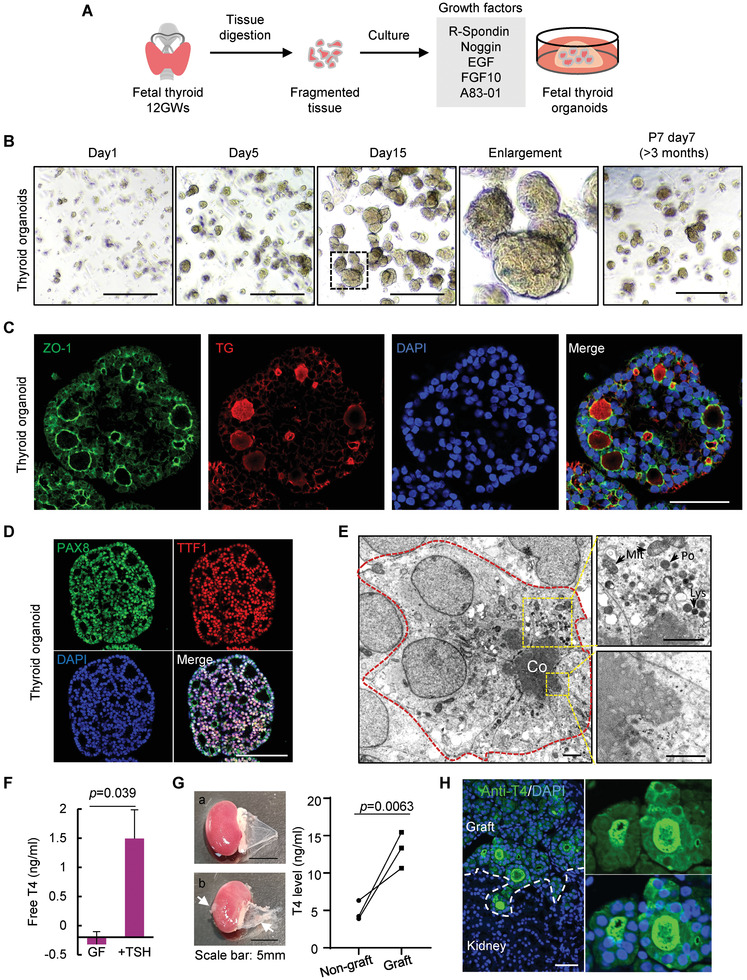
Generation of reproducible human fetal thyroid organoids. A) Schematic depicting the digestion and seeding of fetal thyrocytes, and the culture medium of hFTOs. B) Representative images of a series time points of Passage 0 (P0) and Passage 7 (P7) thyroid organoids. Scale bars = 500 µm. The dotted box indicated zoomed region. C) Immunofluorescence double‐labeled hFTOs (P1) of ZO‐1 and TG, and nuclei with DAPI. Scale bar = 50 µm. D) Immunofluorescence double‐labeled hFTOs (P1) of PAX8 and TTF1 (also known as NKX2‐1), and nuclei with DAPI. Scale bar = 100 µm. E) Transmission EM of hFTOs showing typical monolayer epithelial follicle structures formed by monolayer cells (outline with red dashed line). Co, colloid; scale bar = 5 µm. The upper box showing mitochondria (Mit), lysosome (Lys), and peroxisomes (Po) in cytoplasm, scale bar = 2µm; the bottom box showing the microvilli, scale bar = 1µm. F) Free T4 secretion measured after 4 days culturing with/without TSH simulation in growth factors (GF) medium. Data are represented as mean ± SD in two independent experiments. G) On the left panel: Photos of NSG mouse kidney with stripped renal capsule. a) Non‐graft kidney with smooth renal capsule; b) The grafted kidney with xenograft under the capsule. White arrow indicates xenograft. On the right panel: T4 level of supernatant of renal capsule with/without xenograft detected by ELISA. The wires represent three pairs of kidneys in three independent mice. H) The immunofluorescence of anti‐T4 and DAPI in the grafted tissue. A zoomed‐in photo is showing on the right. White dashed line divided the graft and kidney tissue. Scale bar: 50 µm.

To build a research model for human fetal thyroid development, we were set to establish a culture system for hFTOs in vitro. The advantage of using fetal organ is that it has completed thyroid specification but still remains characteristics of progenitor cells. Thyroid glands from donor fetuses were dissociated and embedded in Matrigel with indicated medium (**Figure** **3**A). To obtain an optimal culture system for fetal thyroid organoids, we started from commonly used conditions for human epithelial organoids, containing R‐spondin1, EGF, Noggin, FGF10, and A83‐01, and then withdrew each of them from the medium to test the importance of these components. We found that R‐spondin1, EGF, FGF10, and A83‐01 were necessary for the primary growth of thyroid organoids (Figure S4A–D, Supporting Information). Although the removal of Noggin had no significant impact on the primary growth of thyroid organoids, it shortened the lifespan of organoids (Figure S4B, Supporting Information). Hence, we retained Noggin with R‐spondin1, EGF, FGF10, and A83‐01 in this growth factors (GF) medium. Effectively, culturing in GF medium sustained hFTOs growth and organoids could be passaged for more than 3 months without morphology changes (Figure 3B).

Follicular organization of thyrocytes is considered the prerequisite for thyroid hormone biosynthesis.^[^ ^21^ ^]^ Our hFTOs displayed the structure of monolayer follicular epithelium, which resembled the original gland structure. By immunofluorescence analyses, we observed that the lumen formed by monolayer follicular cells with apically oriented ZO‐1, and abundant TG that synthesized intracellularly was stored there to constitute the major part of colloid (Figure 3C). Two key thyroid transcription factors, PAX8 and NKX2‐1 that are restrictively co‐expressed in TFCs,^[^ ^22^ ^]^ were readily detected in hFTOs either by immunofluorescent staining or qRT‐PCR (Figure 3D and Figure S4E, Supporting Information). Similarly, mRNA expression of known thyrocyte markers, including *TG*, *TSHR*, and *TPO*, were highly expressed in hFTOs (Figure S4E, Supporting Information).

Detailed follicle morphology of thyroid organoid was further revealed by transmission electron microscope. Figure 3E showed an intact follicle formed by several columnar epithelial cells with high electron density colloid in lumen. These highly polarized cells contained abundant mitochondria and lysosome near the lumen side (Figure 3E, top right) and the apical plasma membrane is furnished with microvilli (Figure 3E, bottom right), consistent with primitive thyroid tissue.^[^ ^23^ ^]^

Importantly, we found that hFTOs had the ability to respond to TSH simulation to produce thyroxine (T4) (Figure 3F). In sum, our results support that our hFTOs recapitulated normal follicular cell characteristics.

### 2.5 hFTOs Preserved Fetal Thyroid Lineages and Retained the Ability to Generate T4 Secreting Thyroid Follicles

The immunofluorescence staining for Ki67 revealed that proliferating cells scattered into the organoid (Figure S4C, Supporting Information), reflecting organoid growth and the proliferative ability of hFTOs in long‐term culture (Figure S4F, Supporting Information). The rate of colony formation had no significant change for at least six passages and the NKX2‐1/PAX8 positive cells could be maintained (Figure S4G,H, Supporting Information). Karyotypic analysis of the sixth passage of hFTOs showed that most cells remained a normal karyotype (Figure S4I, Supporting Information). In addition, cell cryopreservation and resuscitation of hFTOs were conducted (Figure S4J, Supporting Information).

The unique physiological function of thyroid follicular epithelial cells is their capacity to synthesize and secrete thyroid hormones. To assess the potential function of the hFTOs derived from fetus in vivo, we grafted the organoids under the kidney capsules of NSG mice (Figure S5A, Supporting Information). Hematoxylin and eosin (H&E) staining evaluation of the kidneys 7 weeks after transplantation demonstrated successful integration of grafted organoids in the host niche, and numerous follicles formed by monolayered epithelium were present at the renal capsule (Figure S5B, Supporting Information). Immunostaining analyses revealed cytosolic TG expression and TG deposition in the luminal compartment and polarized NIS expression at the basolateral membrane, corroborating the development of mature thyroid follicles at the grafting site (Figure S5C, Supporting Information). The engraftment generated from human‐derived organoids was confirmed by the presence of human NuMA (Figure S5D, Supporting Information). Further detection of thyroid hormone T4 demonstrated the function of hormone secretion of the transplanted thyrocytes (Figure 3G,H). And Ki67 expression indicated the proliferative capacity of the graft (Figure S5E, Supporting Information). Overall, we established a hFTO model, which had the capacity for long‐term culture and gland generation.

### 2.6 cAMP Activation Boosted Thyroid Follicle Maturation for Hormone Secretion in hFTOs

Uncovering the regulation mechanism of hormone production is essential for a better understanding of thyroid development.

**Figure 4:**
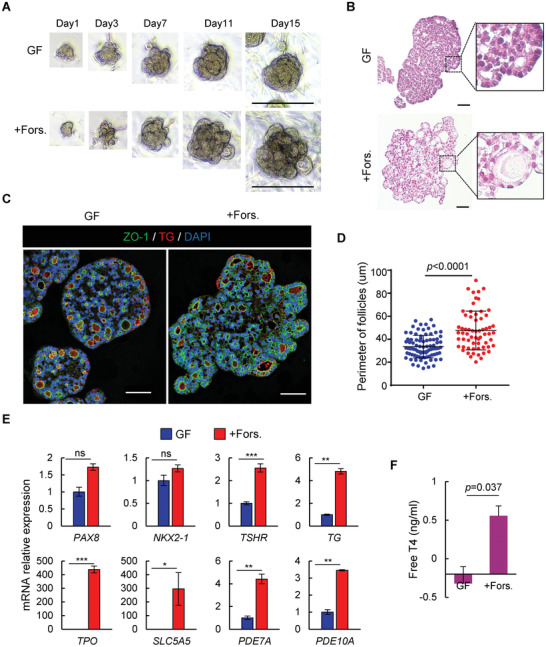
cAMP activation boosted thyroid follicles maturation for hormone secretion in hFTOs. A) Representative bright‐field images of organoids at indicated days during primary culture at growth‐factor (GF) medium and forskolin treatment (+Fors.) condition. Scale bar = 100 µm. B) Hematoxylin and eosin staining on organoids indicated in (A), scale bar = 50µm. C) Immunofluorescence double‐labeled thyroid organoid of ZO‐1 and TG, and nuclei staining with DAPI. Scale bar = 50 µm. D) Quantification of follicle perimeter in organoids that are cultured in growth‐factor (GF) medium and forskolin treatment (+Fors.) condition. Data are represented as mean ± SD, adjusted *p*‐value (Unpaired *t*‐test) *p* < 0.0001. *n* = 87 for GF condition and *n* = 63 for Forskolin treatment condition. E) qRT‐PCR analysis of gene expression of thyrocyte markers and cAMP signaling related genes. F) Free T4 secretion measured after 4 days culturing in growth‐factor (GF) medium and forskolin treatment (+Fors.) condition. Data are represented as mean ± SD in two independent experiments.

We aimed to simulate the maturation process of thyroid development in vitro using our hFTOs. In the KEGG analysis of thyroid single‐cell transcriptome between 12GWs and 16GWs, we found that multiple signaling pathways were activated in 16GWs, including TNF, FoxO, IL‐17, cAMP, and mTOR signaling pathways (Figure 1D). Also, SCENIC analysis showed that a subset of TFs, related to the cAMP signaling pathway, was specifically active in thyroid hormone‐synthesizing TFCs (Figure 2C). cAMP signaling was known as the main mediator for the thyrocyte proliferation and differentiation stimulated by TSH in adults.^[^ ^24^ ^]^ However, cAMP signaling in human fetal thyroid development remains to be explored. Hence, we added forskolin, a cAMP pathway agonist, to the GF medium of hFTO culture. We found that hFTOs formed larger follicular structures in primary culture after 4 days of forskolin treatment (**Figure** **4**A,B). Immunofluorescence double‐labeling of ZO‐1 and TG showed the presence of follicle structures in hFTOs, formed by the monolayer follicular cells with the apical membrane in contact with the colloid of the follicles (Figure 4C). The follicle size was significantly larger in forskolin‐treated organoids in comparison with the untreated controls (Figure 4D). In addition, we found that the sodium–iodide symporter SLC5A5 was highly expressed in forskolin‐treated organoids (Figure S4K, Supporting Information). Consistently, mRNA expression level of thyroid markers *SLC5A5*, *TSHR*, *TG*, and *TPO* were upregulated in forskolin‐treated organoid (Figure 4E). The expression levels of cAMP‐specific phosphodiesterase *PDE10A* and *PDE7A* were also significantly upregulated as expected. Meanwhile, the expression levels of two transcriptional factors *NKX2‐1* and *PAX8*, did not change significantly after treatment (Figure 4E).

To demonstrate that these thyroid organoids have the function of secreting thyroxine, we measured T4 level in the supernatant. After treatment of forskolin for 4 days, T4 secretion was detected and a significant increase was observed in treated organoids, when compared to untreated ones (Figure 4F). The above results suggest that hFTOs retained the ability to undergo further maturation and to produce thyroid hormones in vitro, and the cAMP agonist forskolin is an optional stimulation for this process. Because of its hormone‐secreting function, we hereafter referred the forskolin‐treated organoids as human matured thyroid organoids (hMTOs).

It's worth mentioning that, regulation of the other signaling pathways (e.g., TNF, FoxO, IL‐17, and mTOR) did not alter the gene expression level of thyroid specific markers, although TNF activation changed the morphology of hFTOs to cystic, and mTOR inhibition induced growth arrest of hFTOs (Figure S6, Supporting Information).

### 2.7 cAMP‐Induced hFTOs to hMTOs Transition Recapitulated Human Thyroid Development Ex Vivo

**Figure 5:**
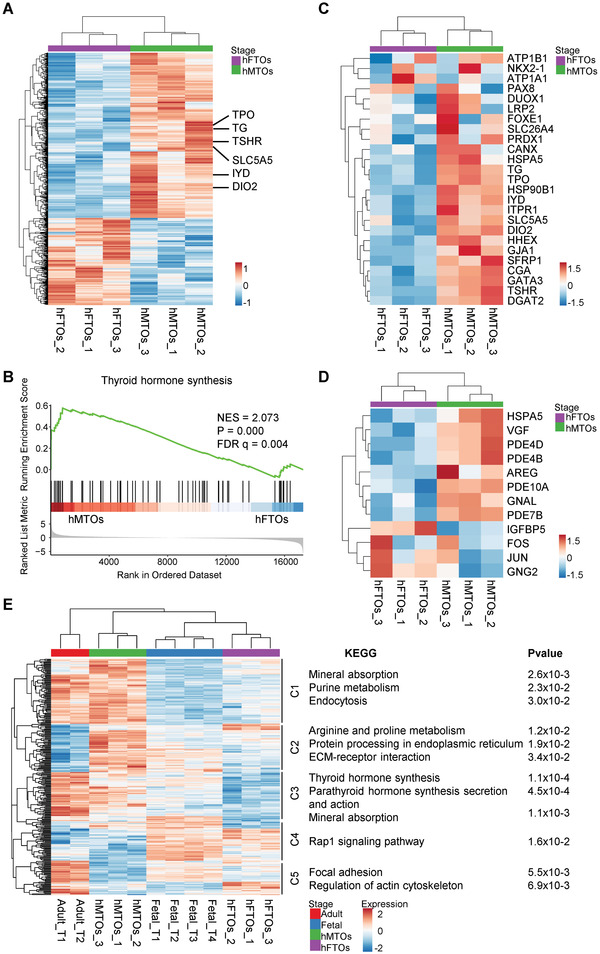
cAMP‐induced hFTOs to hMTOs transition recapitulated human thyroid development ex vivo. A) Heatmap of differentially expressed genes (DEGs; log2fold changes ≥ 0.3, *p*‐value ≤ 0.005) between hFTOs and hMTOs. Three independent experiments were sequenced for each stage. Genes associated with hormone generation were marked out highlighting upregulation of TPO, TG, TSHR, SLC5A5, IYD, and DIO2 in hMTOs. B) GSEA analysis showing enrichment of thyroid hormone synthesis pathway in hMTOs versus hFTOs. C) Heatmap of reported thyroid hormone related genes in hFTOs and hMTOs. D) Heatmap of reported cAMP related genes in hFTOs and hMTOs. E) Heatmap of different stages samples between biological replicates of Adult_Tissues (*n* = 2), Fetal_Tissues (*n* = 4), hFTOs (*n* = 3), and hMTOs (*n* = 3). And the main KEGG‐enriched terms of clusters 1–5 are shown on the right. The color from blue to red indicates the relative expression levels from low to high.

To understand the cAMP‐induced maturation process in established organoids, we assessed global gene expression profiles for hFTOs and hMTOs derived from three independent fetuses by performing RNA‐seq. Using DESeq2, we identified 719 DEGs between hFTOs and hMTOs, including 473 upregulated and 246 downregulated genes (**Figure** **5**A). Prominently, genes involved in hormone generation such as *DIO2*, *TPO*, *IYD*, *TG* and *SLC5A5* were upregulated in hMTOs (Figure 5A). Gene set enrichment analysis (GSEA) of DEGs identified the upregulation of thyroid hormone synthesis signature in hMTOs (Figure 5B). Likewise, we observed upregulated expression of genes associated with thyroid hormone synthesis (Figure 5C and Figure S7A, Supporting Information) and cAMP‐mediated signaling (Figure 5D and Figure S7B, Supporting Information) in hMTOs.

To assess the maturity of hMTOs, we examined the transcriptomes of four pieces of primary fetal thyroid tissue and two adult thyroid gland samples. We found that the DEGs between hFTOs and hMTOs are significantly overlapped with DEGs between Fetal and adult thyroids (see Experimental Section, Fisher's exact test, *p* < 7.375 × 10^−21^). Among the 302 overlapped DEGs, we identified five distinct clusters (C1–C5) via hierarchical clustering (Figure 5E). Genes from C1 and C3 that were upregulated in both hMTOs and adult thyroid were associated with mineral absorption and hormone synthesis and secretion, supporting that hMTOs are closer to adult thyroid gland in function. These results support that the transition from hFTO to hMTO by cAMP induction resembles thyroid development and maturation.

### 2.8 Dissection of Human Thyroid Development by Organoid Atlas

**Figure 6:**
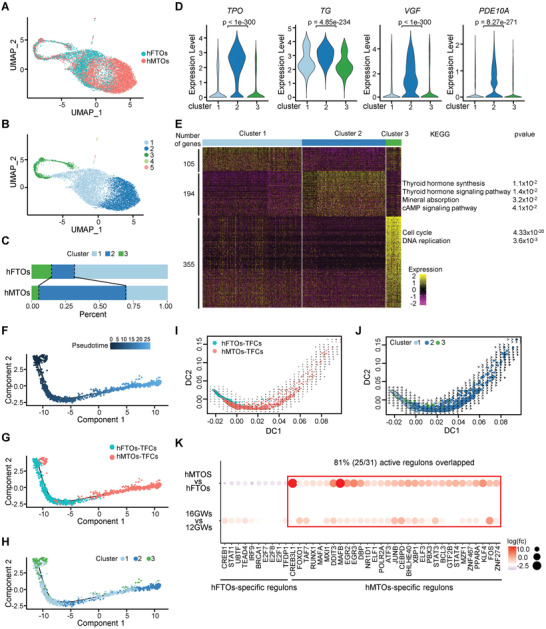
Dissection of human thyroid development by organoid atlas. A) UMAP for scRNA‐seq of thyroid organoids. Blue dots represent cells from hFTOs, and red dots represent cells from hMTOs. B) UMAP showing cell clusters of (A), partitioned by single‐cell transcriptome. C) Bar diagram showing the fraction of clusters 1–3 from thyroid follicular cells of hFTOs and hMTOs. The clusters partitions are shown in (B). D) Violin plot comparing the expression levels of marker genes in clusters 1–3 from thyroid follicular cells of hFTOs and hMTOs. *p*‐value was performed by Wilcoxon Rank Sum test between cluster 2 against other clusters. E) Heatmap and KEGG‐enriched terms of clusters 1–3. The numbers of genes upregulated in each cluster are indicated on the left. The color from purple to yellow indicates the relative expression levels from low to high. F) Ordering of follicular cells from hFTOs and hMTOs along a pseudotemporal trajectory using Monocle2. Blue from deep to light represents the more primitive cellular state to the more differentiated cellular state. G) Position of follicular cells from hFTOs and hMTOs along the pseudotemporal trajectory. H) Position of follicular cells belonging to the three different clusters along the pseudotemporal trajectory. I) Diffusion map showing RNA velocity for TFCs. For each cell, arrows indicate the location of the future cell state. The samples marked by color are the same as (A). J) Diffusion map showing RNA velocity for TFCs. For each cell, arrows indicate the location of the future cell state. The clusters marked by color are the same as (B). K) Dot plot of regulon activity analyzed by SCENIC. The top row represents specific regulons comparing between hMTOs and hFTOs. The bottom row represents 16GWs versus 12GWs. The color from purple to orange indicating logFold Change from low to high and the dot size indicating percentage of cells within sample expression the gene.

To better understand cell type specification during hFTO‐hMTO transition, we performed scRNA‐seq on thyroid organoids. After quality control, 11385 single cells of hFTOs and hMTOs were analyzed and visualized by UMAP (**Figure** **6**A). The UMAP plot revealed five cell clusters with distinct expression of marker genes (Figure 6B). We identified the cells from cluster 1, 2, and 3 as TFCs, based on the co‐expression of thyroid markers and specific transcriptional factors (Figure S8A–C, Supporting Information). Cluster 4 cells were identified as fibroblast cells and cluster 5 cells were likely basal cells. Separation of clusters 1, 2, and 3 suggested heterogeneity of TFCs in organoids. Cell component proportion analysis showed an increase of cluster 2 cells and a decrease of cluster 1 and cluster 3 cells in hMTOs, as compared to hFTOs (Figure 6C). Interestingly, cluster 2 cells displayed upregulation of thyroid hormone synthesis‐related genes, such as *TG* and *TPO* (Figure 6D). In addition, simultaneous upregulation of *VGF* and *PDE10A* indicated a cAMP activation signature. It suggested that cluster 2 cells may execute or impend execute hormone generation function. Totally, we obtained 105, 194, and 355 DEGs in cluster 1, 2, and 3, respectively. KEGG analysis of cluster 2 specific genes showed an enrichment of thyroid hormone signaling and cAMP signaling pathways (Figure 6E). Consistently, UMAP plot showed apparent expression of hormone synthesis‐related genes (*TPO*, *TG*, and *GPX3*) in cluster 2 from hMTOs (Figure S8E,F, Supporting Information). Based on the expression pattern of hormone synthesis genes and the enrichment of thyroid hormone signaling, we defined cluster 2 as the mature‐follicle cell cluster, and cluster 1 as the naïve‐follicle cell cluster.

We identified cluster 3 as the proliferating cells in thyroid organoids, cause DEGs in cluster 3 were enriched in cell cycle and DNA replication pathways (Figure 6E), including cell‐cycle genes such as *TUBA1B*, *HMGB2*, *TOP2A*, and *TUBB* (Figure S8A, Supporting Information). We next determined the cell cycle state of each cell within hFTOs and hMTOs using the “CellCycleScoring” function in Seurat. Consistent with the proliferating cell state, most cells from cluster 3 were at the G2/M phase (Figure S9A, Supporting Information). Using *TOP2A* and *MKI67* labeled proliferating cells, we demonstrated that the decreased proportion of cluster 3 in hMTOs (Figure 6C) corresponded to a decrease in proportion of proliferating cells from 20.8% in hFTOs to 5.9% in hMTOs (Figure S9B,C, Supporting Information). Consistent with the low turnover rate in adult normal thyroid, most of TFCs sustain at the quiescent state. The proportion of proliferating follicular cells, based on our scRNA‐seq data, was 11.0% in fetal thyroid tissue and 0.2% in adult thyroid tissue, according to previously reported scRNA‐seq data^[^ ^25^ ^]^ (Figure S9B,C, Supporting Information). Moreover, immunofluorescent staining of Ki‐67 also showed a dramatic decrease of proliferating cells in hMTOs, as compared to hFTOs (Figure S9D,E, Supporting Information). In the meanwhile, the ratio of follicular cells to nonfollicular cells increased in hMTOs (Figure S9F, Supporting Information). Therefore, hMTOs were deemed more similar to adult follicular cells at the cell cycle level.

Pseudotemporal ordering of TFCs from hFTOs and hMTOs using Monocle2 was then applied to shed light on the cell hierarchy of thyroid cells’ developmental process. We plotted a trajectory from hFTOs to hMTOs (Figure 6F,G). Cells from the proliferating cluster 3 were placed at the start of the pseudotime axis, that may be considered the progenitor state while hormone synthesis related cells from cluster 2 were placed at the end (Figure 6H). The heatmap showed the dynamic expression of genes ordered by pseudotemporal expression pattern (Figure S8G, Supporting Information). Gene sets 4 and 5 located at later pseudotemporal trajectory contained genes associated with the thyroid hormone signaling pathway (e.g., *TG, TPO, TSHR, SLC26A4, DUOXA2, DUOX1, IYD*, and *CANX*) and the cAMP signaling pathway (e.g., *JUNB, SOX9, PDE10A*, and *PDE4B*) (Figure S8G, Supporting Information). RNA velocity analysis suggested that the cells from hFTOs were more located at the root of the differentiation hierarchy, whereas the cells from hMTOs were more located at the endpoint (Figure 6I), and the trajectory of differentiation evolved from clusters 1/3 to cluster 2 (Figure 6J). Quite a few regulons inferred by SCENIC were active only in hMTOs, comparing to hFTOs (Figure 6K). Importantly, we found that 81% (25/31) of hMTO‐specific TF regulons were also active in 16GW‐fetal thyroid tissue (Figure 6K). Collectively, the activation of cAMP in fetal thyroid organoids drove the maturation of fetal TFCs, a process resembling the development of fetal TFCs, indicating that fetal thyroid organoids system can be used as a model for studying thyroid development.

### 2.9 Enhanced Chromatin Accessibility Permitted Transcriptome for Fetal Thyroid Fate Determination

**Figure 7:**
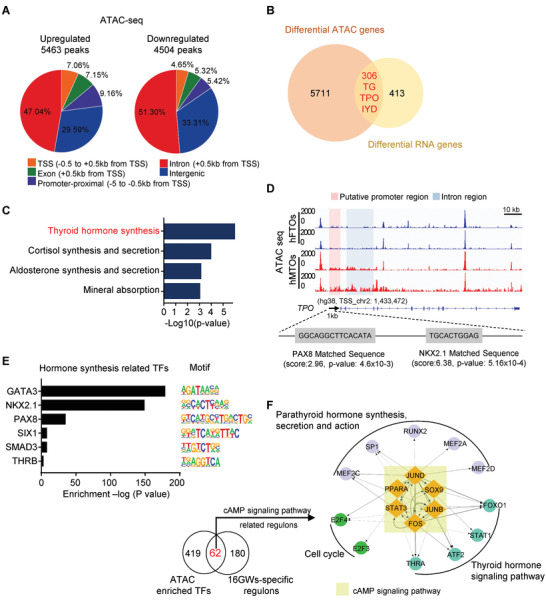
Enhanced chromatin accessibility permitted transcriptome for fetal thyroid fate determination. A) Distribution of differentially accessible ATAC peaks on genome in hFTOs and hMTOs. B) Venn diagram showing the overlap between differential peak genes from ATAC‐seq and differential expressed genes from RNA‐seq. The comparison was between hFTOs and hMTOs. C) KEGG analysis of upregulated overlap genes (220/306 genes), showing enrichment of thyroid hormone synthesis pathway. D) ATAC‐seq signals at TPO genome site of two biological replicates in hFTOs and hMTOs showing by integrative genomics viewer. PAX8 and NKX2.1 motif scanning at TPO genome site using MEM Suite website. Pink framework indicates putative promoter regions within 1 kb from TSS (transcription start site), and blue framework indicates intron regions. Scale bar = 10 kilobase (kb). E) Motif analysis of differential ATAC peaks in hMTOs upregulated genes for putative transcription factor (TF) binding sites of thyroid hormone synthesis related using HOMER Database. F) Left: Number of overlapped TFs between ATAC‐enriched TFs and 16GWs‐specific regulons. Right: Regulatory networks of selected TFs (nodes) from motif analysis of differential ATAC peaks. Arrow on edge from node X to node Y indicates that TF‐X regulates TF‐Y. To aid interpretation of the network, nodes are grouped and colored according to their regulatory signaling pathways.

To determine the dynamics of open chromatin landscape that underlie the profound transcriptomic differences, we carried out ATAC‐seq for two biological replicates of hFTOs and hMTOs. By analyzing the ATAC‐seq data, we identified 119 848 accessible chromatin regions (indicated by ATAC peaks) in hFTOs and 119 744 accessible chromatin regions in hMTOs (Figure S10A, Supporting Information). Further analysis revealed that induced maturation led to 9967 significantly changed peaks (5463 sites opened and 4504 sites closed) in chromatin accessibility, with more open sites accumulated in promoter proximal and TSS (9.16% + 7.06%) regions than close sites (5.42% + 4.65%) (**Figure** **7**A). Chromatin accessibility at promoter proximal and TSS regions directly influences gene transcription. Hence, we compared the differential peak genes from ATAC‐seq with 719 DEGs identified by RNA‐seq, gathering 306 overlap genes, including *TPO*, *TG*, and *IYD* (Figure 7B). Among them, 220 genes were upregulated within 306 overlap genes and they were enriched in thyroid hormone synthesis pathway by KEGG analysis (Figure 7C).

Using integrative genomics viewer, highly reproducible results of biological replicates showed increased accessibility at putative promoter and intron regions at the *TPO* locus in hMTOs (Figure 7D), consistent with this gene's upregulated transcriptional level (Figure 5C). Motif enrichment assay revealed that the top motifs of increasing accessible chromatin region corresponded to high‐affinity binding TFs, which were involved in thyroid gland development/thyroid hormone generation, including pivotal regulators GATA3, NKX2‐1, and PAX8 (Figure 7E). Studies had demonstrated that the core binding sequences of transcriptional factors PAX8 and NKX2‐1 overlap in the *TG* and *TPO* promoters to drive transcriptional activation of these genes.^[^ ^26^ ^]^ By motif scanning using MEM Suite, we identified ATAC‐seq peaks at potential PAX8 and NKX2.1 binding sites in the promoter regions of *TPO* and *TG* (Figure 7D and Figure S10B, Supporting Information), suggesting that the regulation of these two transcriptional factors on *TPO* and *TG* expression during thyroid development were also recapitulated in our organoid model.

TF has the function of gene regulation and groups of TFs are vital for many important cellular processes. To determine the extent to which hMTOs maturation recapitulate the TFs regulation of human fetal thyroid development, we analyzed the overlapped TFs between ATAC‐enriched TFs and 16GWs‐specific regulons (Figure 7F). We observed that quite a few TFs are shared between these two compared datasets, which is a strong indication of the reliability of resemblance. To further probe these overlapping TFs, we reconstructed a picture of the TF regulatory networks. There we found a group of cAMP signaling pathway related regulons that displayed predicted regulation in thyroid hormone signaling pathway, indicating their importance in regulating thyroid development (Figure 7F). Taken together, we propose that increasing chromatin accessibility is important to permit transcriptome for fetal thyroid fate determination, and indicate that organoid system recapitulates the developmental process of fetal thyroid to a great extent.

## 3 Discussion

Here, we reported a genome‐wide transcriptome view of human thyroid development at single‐cell resolution, and we established an organoid model system to recapitulate human thyroid development. The key conclusions of this study are that 1) we revealed the critical time window for thyroid development where cell composition changes with the human thyroid developmental process, and a subpopulation of follicular cells is mainly responsible for hormone generation; 2) human fetal tissue‐derived thyroid organoids largely recapitulated the characteristics of fetal thyroid, with potentialities for long‐term culturing and transplantation; and 3) hFTOs is a suitable model for the real‐time monitoring of fetal thyroid development, which could be induced for further maturation that well mimic the human thyroid development process.

Through single‐cell atlas, we expounded heterogeneity among thyroid epithelial cells and multiple signaling pathways (including the cAMP signaling pathway) that coregulated thyroid gland development. cAMP activation is essential for thyroid proliferation and differentiation.^[^ ^27^ ^]^ Several studies have demonstrated that cAMP is critical for thyroid maturation in human‐^[^ ^13^, ^28^ ^]^ and mouse‐derived model.^[^ ^29^ ^]^ Transcriptomic signature comparison of human early embryonic thyroid and adult thyroid also identified cAMP signaling upregulation in adult thyroid.^[^ ^11^ ^]^ Consistently, the dependence of adult TFC cells on the cAMP pathway has been reported by the latest study on adult mouse and human thyroid organoids.^[^ ^30^ ^]^ However, when cAMP initiated in fetal thyroid and when subsequent cascade events occur in the narrow time window from thyroid organogenesis to functional maturation still remain elusive. Our study indicates the significance of cAMP activation during fetal thyroid maturation and demonstrates heterogeneity of fetal thyrocytes in cAMP signal response. In classical physiology, TSH binds to a specific TSH receptor (TSHR), which belongs to the family member of GPCRs, to activate adenylate cyclase and increase cAMP levels in thyrocytes. In this study, we found other GPCRs which may be associated with thyroid maturation. Hence, a more detailed understanding of the genes co‐expressed with those of cAMP would be beneficial for further study. In addition, other specific signals are reported to be involved in modulation of proliferation and differentiation in human thyrocyte, such as Wnt^[^ ^31^ ^]^ and mTOR.^[^ ^32^ ^]^ Ligand‐receptor interaction network of thyroid homeostasis in zebrafish has been shown.^[^ ^33^ ^]^ Further research is expected to provide a full understanding of how these signal pathways integrate with cAMP to regulate thyroid development, and how ligand‐receptor coregulate thyroid maturation in human fetus.

Meanwhile, a portion of candidate regulons were identified during fetal thyroid development from 12 to 16 GWs. Interestingly, most of them were active only at the differentiated state (cluster 3) (Figure 2C). They probably contributed to thyrocyte differentiation and hormone production. On the other hand, *YY1*, which was reported that plays a positive role in regulating and maintaining the pluripotency of mammalian cells,^[^ ^34^ ^]^ was restrictively expressed in the sample of 12GWs. Histological comparison of 16GWs and 12GWs showed that candidate transcriptional factors (FOXO1, MAFB, and STAT3) were highly expressed in later GWs (Figure 2F), suggesting that candidate regulons could reflect the developmental state to some extent. Definitely, a few endothelial cells or stromal cells in 16GWs’ thyroid tissue were also STAT3 positive. This might be due to microenvironmental signals of a diverse array of cytokines, GF, and peptide hormones. As STAT3 plays distinct roles in regulating gene programs of epithelial, endothelial, immune, and stromal cells,^[^ ^35^ ^]^ the mechanism of how STAT3 activation in thyroid maturation determines the cell fate of TFCs and other cell types remains to be answered. Therefore, this study gives valuable information for further improvement of thyroid development research. Still, our study has several limitations. Research of this specific narrow time window (12GWs–16GWs) is unable to reveal genes implicated in thyroid dysgenesis, because the gene mutation causing thyroid dysgenesis may occur earlier than the onset of thyroid differentiation.^[^ ^6a^ ^]^ Samples from earlier and later GWs could provide more information on thyroid development.

Because there are currently no human cell lines available that can act as thyroid progenitor cells, the emergence of organoids that display self‐assembling ability in vitro and potential in vivo function, has caught much attention. The in vitro model system based on the directed differentiation of human and mouse derived PSCs/ESCs into thyroid progenitors and mature follicular organoids,^[^ ^13a,b^ ^]^ as well as adult tissue derived organoids^[^ ^13c,d^ ^]^ and cancer‐derived organoids^[^ ^36^ ^]^ have been reported. Considering the fidelity of thyroid specialization and the time window of developmental events, we established a human fetal thyroid‐derived organoids (hFTOs) system. We applied our hFTOs to simulate the maturation of thyroid gland and demonstrated that they recapitulate the follicular structures and hormone production function in vitro. Better yet, treatment with the chemical compound forskolin induced elevation of T4 level in organoids (Figure 4F), which is similar to the result of direct simulation of TSH (Figure 3F). It is noteworthy that EGF stimulation promotes fetal thyroid organoid growth without seemingly a negative impact on differentiation. This is at difference to adult thyroid cells for which EGF is a strong dedifferentiating factor.^[^ ^28^ ^]^ This differential response indicates that models derived from fetal or adult thyroid cells may be suitable for particular research issues. In general, our system makes it possible to monitor dynamic changes during fetal thyroid development. Furthermore, hFTOs might also be useful for gene manipulation which are focused on the exploration of thyroid physiology or pathology, such as gene manipulation in liver organoids that introduced oncogenes to model liver cancer initiation.^[^ ^37^ ^]^ For example, it is well known most congenital hypothyroidism is caused by thyroid dysgenesis, but little is known about the etiology in more than 95% cases.^[^ ^6^, ^38^ ^]^ Several mutations have been found to be associated with thyroid dysgenesis, and this provides an important entry point for further investigation. These include *PAX8*, *NKX2‐1*, *FOXE1*, *NKX2‐5*, *JAG1*, and *GLIS3* genes.^[^ ^39^ ^]^ We suppose the thyroid organoid, derived from first trimester tissues, may help to establish a congenital hypothyroidism model by gene manipulation, and this may facilitate further understanding of thyroid development and the study of congenital thyroid disorders. Further, modification of coculture system with stromal cells, immune cells, and vascular endothelial cells could simulate a more realistic microenvironment of thyroid gland in vitro.

It is worth mentioning that although it is advocated to monitor maternal thyroid hormone level during pregnancy, the risk of current clinical guidelines treatment on mothers is definitely there, that slightly overtreatment of maternal hypothyroidism may lead to fetal developmental disorders.^[^ ^40^ ^]^ Moreover, it remains unclear if the high incidence of thyroid diseases in children and adolescents is associated with fetal thyroid dysplasia. Consequently, it is crucial to deconstruct the developing fetal thyroid and establish a model for recapitulating fetal thyroid development, which is helpful for the study of thyroid development and pediatric disease progression.

In general, our study highlights the novel possibility of fetal thyroid organoids for the research of thyroid development. And we established a potential system for a better understanding of the physiology and signaling pathways of the human thyroid functions and for the development of medical interventions in the field of thyroidology.

## 4 Experimental Section

### Informed Consent

Human fetus collection for the study was approved by the Institutional Ethical Committee of Obstetrics and Gynecology Hospital of Fudan University and the approval number was 2018‐77. All fetuses were obtained with the informed consent by the patient who had made a legally elective abortion from the Obstetrics and Gynecology Hospital of Fudan University. Informed consent confirmed that the patients voluntarily donated fetus for research on human embryonic development with no financial payment. The thyroid glands were obtained from 12–16 GWs healthy fetuses. The research abides by the Declaration of Helsinki principles.

### Tissue Collection and Preparation

The fetal thyroid gland was excised from a donor fetus. The sizes and structures of single lobe of the fetal thyroid at 12GWs and 16GWs were shown by sections with H&E staining, respectively (Figure S1, Supporting Information). The thin layer of connective tissue enveloped on the surface of the thyroid gland was cleaned up under a stereomicroscope, followed by minced and digested process. The fragmented thyroid tissue was subjected to a freshly prepared digestion solution with 10 U mL^−1^ Dnase I (Roche, 04716728001), 100 U mL^−1^ collagenase type I (Thermo, 17100017), and 100 U mL^−1^ collagenase type II (Thermo, 17101015) for 30 min at 37 °C. The digestion solution was pipetted up and down and then filtered through a 100 µm Cell Strainer. After centrifugation of the filtrate at 200 × *g* for 5 min, the supernatant was discarded and prewarmed Red Blood Cell Lysis Buffer was added (Solarbio, R1010) for 3–5 min at 37 °C to remove erythrocytes followed by DPBS washing. For single‐cell sequencing, the cell lysate was dissociated with digestion solution for another 20 min until most cells were dissociated into single cells, which could be confirmed under the microscope. For organoid establishment, pellets/follicles were collected and washed in Advanced DMEM/F‐12 (Invitrogen) three times and then embedded in Matrigel (Corning). For bulk RNA‐seq, pellets/follicles were collected and washed with DPBS, then subjected to RNA extraction using the RNAprep Pure Micro Kit (Tiangen Biotech).

### Fetal Thyroid Organoid Culture and Maturation

The fetal thyroid organoid culture medium consisted of Advanced DMEM/F‐12 supplemented with penicillin–streptomycin (Invitrogen), HEPES buffer (10 mm, Gibco), B27 supplement (1×, Invitrogen), GlutaMax (1×, Invitrogen), *N*‐acetyl‐L‐cysteine (1 mm, Sigma‐Aldrich), retinoic acid (50 ng mL^−1^, MCE), and GFs mentioned in the figures, R‐spondin1 (200 ng mL^−1^, OrganRegen), Noggin (100 ng mL^−1^, OrganRegen), EGF (50 ng mL^−1^, SinoBiological), FGF 10 (100 ng mL^−1^, OrganRegen), and A83‐01 (500 nm, Tocris). 10 µm Y‐27632 (Sigma‐Aldrich) was added for primary culture. Forskolin (10 µm, Selleck, S2449) or TSH (0.3 mIU mL^−1^, Sigma, T9265) were added for stimulating maturation. Fetal thyroid organoids were passaged every 10–14 days. To avoid the influence of passage, the authors used the organoids from passage 2 to passage 3 for RNA‐seq and ATAC sequencing. To prepare frozen stocks, organoids were mixed with Cell Culture Freezing Medium (Gibco) and frozen following standard procedures. Light microscopy images were captured with an Olympus FV3000 camera.

### T4 Analysis

The fetal thyroid organoids were cultured in expansion medium and maturation medium for 4 days, respectively. Organoids were collected and centrifugated for 5 min at 1000 rpm. Supernatant were taken for measuring T4 level by ELISA kit (Biovision) according to the manufacturer's instructions.

### RNA Extraction and Quantitative Real‐Time PCR

For total RNA preparation, organoids of the 24‐well plate were collected and RNAs were extracted and purified using RNAprep Pure Micro Kit (Tiangen Biotech). Purified RNAs were reverse‐transcribed into cDNAs with Reverse Transcription System (Promega), according to the manufacturer's instruction. Quantitative real‐time PCR was performed on CFX384 Touch System (BioRad) using TaqMan Universal PCR Master Mix (Bimake B21202). Expression levels were normalized to the reference gene GAPDH, and the primers of target genes were listed in Table S2, Supporting Information.

### Histology and Immunohistochemistry

For the H&E or immunohistochemical staining, tissues were fixed overnight in 4% paraformaldehyde and processed for paraffin embedding. The thyroid organoids were collected gently and fixed in 4% PFA solution for 2 h, then embedded in 3% agarose gel before paraffin embedding process. Subsequently, 5‐µm‐thick sections were mounted on slides.

After deparaffinization and rehydration, sections were subjected to antigen retrieval and endogenous peroxidases were quenched, and then blocked with 10% BSA in PBS for 1 h at room temperature. Primary antibodies were then applied at the appropriate dilutions and incubated overnight at 4 °C in a humidified chamber. Next, the biotinylated secondary antibodies *α*‐mouse IgG or *α*‐rabbit IgG (Invitrogen, 1:500) was added and incubated for 1 h, followed by the detection with streptavidin‐HRP and DAB chromogen (Invitrogen) according to the manufacturer's recommendations. Finally, slides were counterstained with Mayer's hematoxylin, dehydrated, and mounted. Images were taken by cellSens system of Olympus microscope.

### Immunofluorescence

Cultured thyroid organoids were collected and fixed in 4% paraformaldehyde for 30 min at 4 °C, washed with PBS, and permeabilized with 0.25% Triton X‐100 (Sigma) in PBS for 10 min. The organoids were then washed with PBST (PBS containing 0.1% Tween 20) and blocked by 5% BSA in PBST for 1 h at room temperature. Organoids were incubated with the primary antibodies at 4 °C overnight, washed with PBST for three times, and then incubated with the secondary antibodies and Hoechst for 1 h at room temperature in the dark. Organoid imaging was performed on confocal microscope (OLYMPUS, FV3000). The primary and secondary antibodies used for immunostaining are listed in Table S2, Supporting Information.

### Colony Formation Assay

hFTOs maintained in culture medium for around 10 days (reaching a density of 60–70%) were dissociated into single cells using TrypLE Express. 1000 cells were plated per 48‐well and further cultured in the culture condition. The number of organoids was counted at around days 10–14. Each experiment was performed with three wells of 48‐well plate, and two biological replicates were tested.

### Karyotyping

Due to the low turnover rate and the spheroid formation of thyroid organoids at 3D culture, the authors switched the organoids to transitory 2D culture to allow better contact with nocodazole, a mitosis blocking drug. The late (sixth) passaged organoids were harvested at day 10–14 in culture, then dissociated into single cells and cultured in 2D condition. 24 h later, 1 mg mL^−1^ nocodazole was added and treated for 20 h, then processed with standard karyotyping protocol. In detail, cells were digested with 0.25% trypsin, then collected and incubated in 0.075 m KCl for 20 min at 37 °C. The cells were then fixed with Carnoy's Fixative (3:1 ratio of methanol: glacial acetic acid) for 15 min, for three times in total. After that, cells were dropped onto iced slides and air‐dried, followed by staining with Hochest for 15 min before mounting.

### Transmission Electron Microscopy

Organoids in conditional medium were collected and washed twice with PBS, fixed with 2.5% glutaraldehyde for more than 2 h, and then prepared for transmission electron microscopy by Servicebio Company. Briefly, the fixed sample was washed three times with 0.1 m phosphate buffer then postfixed with 2% osmium tetroxide, dehydrated, and cleared in acetone and infiltrated in fresh 100% resin, successively. The samples were then polymerized at 60 °C for 48 h. All of these procedures were performed in 1.5‐mL tubes after the organoids sedimented to the bottom of the tubes. Ultrathin sections (70 nm) were then prepared, stained with uranyl acetate and lead citrate. Images were captured with a transmission electron microscope (Hitachi) at 80 kV.

### Mouse Xenograft Model

6 weeks NSG (NOD‐Prkdcscid Il2rgem1/Smoc) mice were brought from Shanghai Model Organisms. All the animal experiments were approved by the Institutional Animal Care and compliant with all relevant ethical regulations regarding animal research. For organoids preparation, three 24‐wells of organoids (about 1 × 10^6^ cells) were collected, centrifuged at 200 × *g* for 3 min to remove the supernatant, and resuspended in a 20 µL mixture of 50% Matrigel and 50% medium. Organoids were kept on ice until kidney capsule transplantation surgery.

Mice were anesthetized with pentobarbital sodium (40 mg kg^−1^, i.p.). The lateral site of the left abdomen was shaved, scrubbed with iodine, and a 2‐cm incision of the skin, muscle, and peritoneum was made vertically to subcostal. Cells were injected under the kidney capsule of the exposed left kidney using an insulin syringe. The incision in the peritoneum and skin was sutured and sterilized. 7 weeks later, the grafted mice were sacrificed for histological examination of the kidneys. For functional testing of the graft, the authors took the whole transplanted kidney and the untransplanted kidney (as a negative control), and used surgical forceps dissecting renal capsule to expose the graft. The graft together with the renal capsule was ground in 50 µL cold PBS. After centrifugation at 2000 rpm for 10 min, the supernatant was taken for T4 hormone detection using T4 ELISA kit (Biovision). The animal ethics (2020JS038) was approved by Laboratory Animal Center, School of Life Sciences, Fudan University.

### Statistical Analysis

Student's *t*‐test or one‐way ANOVA test was employed to analyze the parametric experimental results. All *p*‐values were two‐sided and were considered significant only if *p* < 0.01 to account for multiple testing. GraphPad Prism software was used for statistical analysis.

### RNA Sequencing Analysis

Total RNA of organoids, fetal, and adult thyroid tissue were isolated using the RNAprep Pure Micro Kit (Tiangen Biotech) and then sent to the company (Berry Genomics) for quality control and sequencing. RNA‐seq libraries were prepared with the Illumina HiSeq platform and more than 40 million reads were obtained for each sample. The raw sequencing data quality was checked by FastQC (https://www.bioinformatics.babraham.ac.uk/projects/fastqc/). STAR 2.7.1a was then used to align reads to human genome (http://labshare.cshl.edu/shares/gingeraslab/www‐data/dobin/STAR/STARgenomes/Human/GRCh38_Ensembl99_sparseD3_sjdbOverhang99/) with default parameters. Bam files were sorted by Samtools and count matrices were generated by featureCounts v2.0.1.

Downstream analysis was processed in R v4.0.3. Briefly, DESeq2 v1.30.0 was used to identify DEG. Genes with *p*‐value ≤ 0.005 were regarded as DEGs. ClusterProfiler v3.18.0 was used to do GSEA and gene expression heatmaps was generated by pheatmap v 1.0.12.

### ATAC‐Seq Analysis

Organoids were digested into single cells and underwent FACS to capture live cells (1.0 × 10^4^). The ATAC‐seq sample was prepared following three major steps as in:^[^ ^41^ ^]^ briefly, to prepare nuclei, the authors used cold lysis buffer (10 mm Tris‐HCl (pH 7.4), 10 mm NaCl, 3 mm MgCl2, 0.5% NP‐40); immediately treated with Tn5 transposase (Vazyme Biotech) for 30 min transposition reaction at 37 °C, then directly purified using clean beads (Vazyme Biotech); at last, amplified library fragments using Index Kit V2 for Illumina (Vazyme Biotech). Constructed library was sequenced on Illumina HiSeq platform by Berry Genomics. PEPATAC pipeline (http://pepatac.databio.org/en/latest/) was followed to process ATAC‐seq data. Briefly, raw sequencing reads were first trimmed to remove adapters using Skewer v0.2.2, then FastQC was used to validate proper trimming quality. Bowtie2 v2.3.4.3 was used to remove reads mapping to chrM (revised Cambridge Reference Sequence) and repeat regions. After removing these reads, Bowtie2 was used to align the remaining reads to hg38 human genome. Samtools was used to sort and isolate uniquely mapped reads and Picard was used to remove duplicates with MarkDuplicates tools. The authors performed peak calling by MACS2 v2.1.2 with parameters “‐f BAMPE –shift ‐75 –extsize 150 –nomodel –call‐summits –nolambda –keep‐dup all ‐q 0.05.” To find significantly changed peaks, R package DiffBind v3.0.1 was used to perform Statistical test with edgeR method at a threshold of *p*‐value < 0.05. ChIPseeker v1.26.0 was used for peak annotation. Homer v4.11.1 was used to perform motif analysis. MEME Suite v5.3.3 was then used for motif scanning.

### Single‐Cell RNA Sequencing and Data Preprocessing

*10x genomics library preparation*: Dissociated processes of single cells from fetal thyroid gland or organoids were mentioned above. Single‐cell sequencing was performed using 10× Genomics platform. The 10× Genomics libraries were sequenced as 150‐bp paired‐end reads on the Illumina HiSeq 4000 platform.

*Preprocessed data quality control and normalization*: Sample demultiplexing, barcode processing, and UMI counting were completed using the official 10× Genomics pipeline Cell Ranger v 4.0.0. Briefly, adapter sequences were trimmed from 3′ end of the reads. After trimming, the command “cellranger count” was used to generate a gene expression matrix for each library. During this step, STAR was used to align reads to hg38 human genome with default settings. Raw gene expression matrices were loaded into R using the Seurat v3.2.2. To exclude poor quality cells that might result from multiplets or other technical noise, cells were removed that had abnormal high unique feature counts or mitochondrial genes expressed. For the remaining cells, gene expression matrix was first normalized to total cellular read count using a global‐scaling normalization method “LogNormalized,” which was then multiplied by a scale factor of 10 000. After normalization, the UMI count data were log‐transformed.

*Batch effect correction and cell clustering*: To eliminate batch effects between libraries, Seurat standard integration pipeline was followed. Before dimensional reduction, the authors applied scaling function on variably expressed genes that were identified from integration steps. Based on these genes, the dataset was subjected to principal component analysis and the first 30 components were further processed using “TSNE” or “UMAP” function in Seurat. To cluster the cells, a graph‐based unsupervised clustering method with “FindClusters” function was performed.

*Trajectory inference*: To define the development trajectory within data, RNA velocity analysis and pseudotime analysis were applied. For pseudotime analysis, the authors obtained subsets and got raw counts from Seurat object to load into monocle v2.18.0. Then differential genes were computed to use as ordering genes. They chose “DDRTree” as the reduction method and plotted the cells alongside the trajectory with the “plot_cell_trajectory” function. For RNA velocity analysis, they followed the steps of velocyto v0.17. First, they got spliced/non‐spliced RNA molecules using “velocyto run” function. Then they performed the “RunVelocity” function with parameters “deltaT = 1, kCells = 25, fit.quantile = 0.02”. Finally, they used diffusion map which was generated by the “DiffusionMap” function in R package destiny v3.4.0 to show the embedding of arrows.

*Transcription factors’ regulon activity determination*: To investigate the genetic network, the authors used R package SCENIC v1.2.4 to perform single cell regulatory network inference and clustering. Following the standard pipeline, they loaded the gene expression matrix with gene symbols as row names, and cell barcodes as column names into SCENIC. Genes were filtered according to RcisTarget database and hg38 was chosen as the default reference genome. Then the co‐expression network was built with GNEIE3, followed by “runSCENIC” to generate the gene regulatory network. Regulon activity was scored with AUCell. Clustering and dimensional reduction were run on the regulon activity.

*Analysis of KEGG differentiation pathways and cell trajectory*: The authors used Wilcox rank‐sum test to identify marker genes of different clusters and ClusterProfiler v3.18.0 to perform GO and KEGG analysis.

## Conflict of Interest

The authors declare no conflict of interest.

## Author Contributions

J.L. and J.Q. contributed equally to this study. J.L. and B.Z. conceived the study; J.L., J.Q., and L.Y. performed the experiments; X.L., X.W.(Xiaoyue), and B.Z. supervised the work; X.C. and X.W.(Xiaoning) contributed to the discussion of the results; and J.L., X.W.(Xiaoyue), and B.Z. wrote the manuscript.

## Supporting information

Supporting Information

## Data Availability

The data that support the findings of this study are available from the corresponding author upon reasonable request.
