# Supplementary material for: Modeling Human Thyroid Development by Fetal Tissue‐Derived Organoid Culture
Source: Adv Sci (Weinh). 2022 Jan 22;9(9):2105568. doi: 10.1002/advs.202105568 (PMC8948548; doi:10.1002/advs.202105568)
Supplement: Supplementary file 1 — Supporting Information [file ADVS-9-2105568-s001.pdf]

ADVANCED SCIENCE
Open Access

Supporting Information

for *Adv. Sci.*, DOI 10.1002/advs.202105568

Modeling Human Thyroid Development by Fetal Tissue-Derived Organoid Culture

Jianqing Liang, Jun Qian, Li Yang, Xiaojun Chen, Xiaoning Wang, Xinhua Lin, Xiaoyue Wang* and Bing Zhao**

Supporting Information

for *Adv. Sci.*, DOI: 10.1002/advs.202105568

Modeling human thyroid development by fetal tissue-derived organoid culture

Jianqing Liang[1], Jun Qian[2], Li Yang[1], Xiaojun Chen[3], Xiaoning Wang[4], Xinhua Lin[1,], Xiaoyue Wang[2,*] and Bing Zhao[1,*]*

Supporting Information

Modeling human thyroid development by fetal tissue-derived organoid culture

Liang et al

Figure S1-S10

Table S1-S3

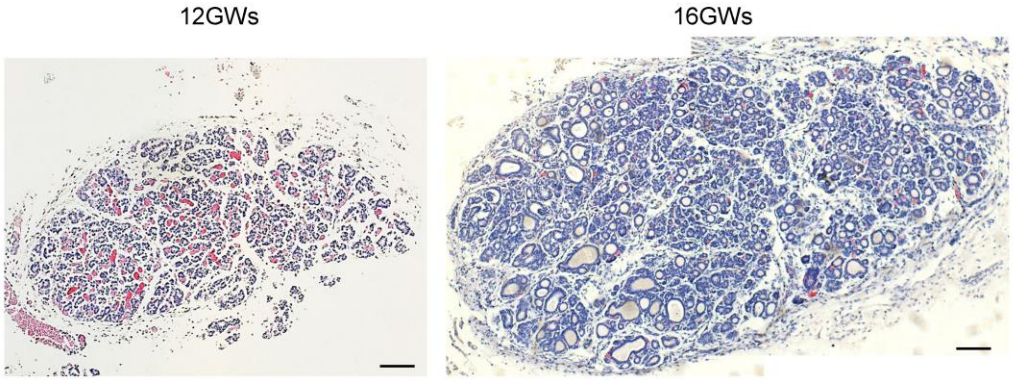

Figure S1. Hematoxylin and eosin staining of single lobe of the fetal thyroid at 12GWs and 16GWs. Related to Figure 1. Scale bar: 100µm.

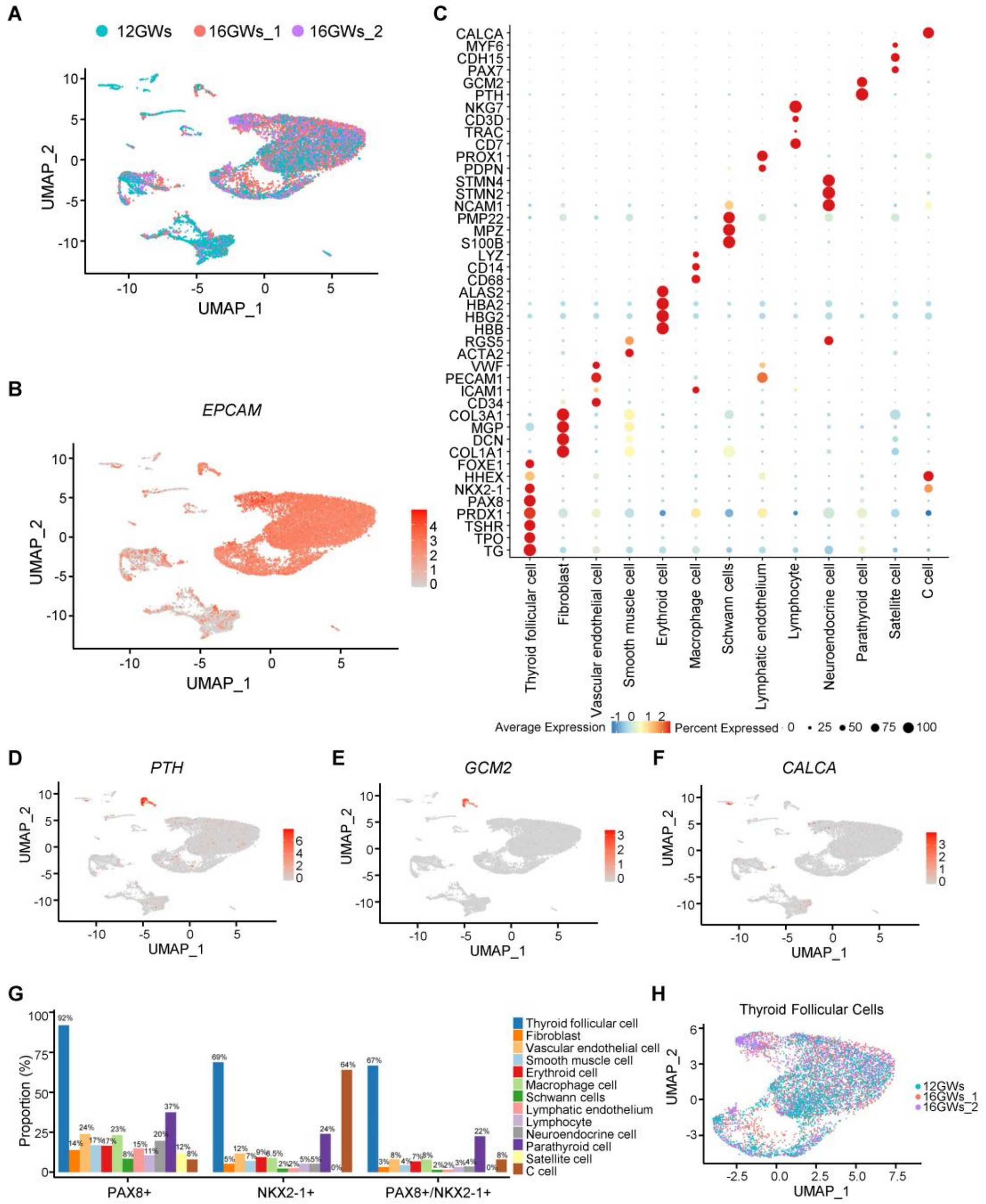

Figure S2. Cell type identification in human fetal thyroid gland by known markers. Related to Figure 1.

(A) UMAP of thyroid glands from three human fetuses: 12GWs, 16GWs_1 and 16GWs_2. (B) UMAP showing the expression levels of EPCAM. (C) Dot plot for known markers of cell types, with color indicating average expression within clusters and dot size indicating percentage of cells within cluster expressing the indicated gene. (D-F) UMAP showing the expression levels of PTH, GCM2 and CALCA. PTH and GCM2 are markers of parathyroid. CALCA is a specific marker of C cell. (G) The proportion of cells with PAX8 or/and NKX2-1 expressed in each defined cell type. (H) UMAP of thyroid follicular cells from 12GWs, 16GWs_1 and 16GWs_2.

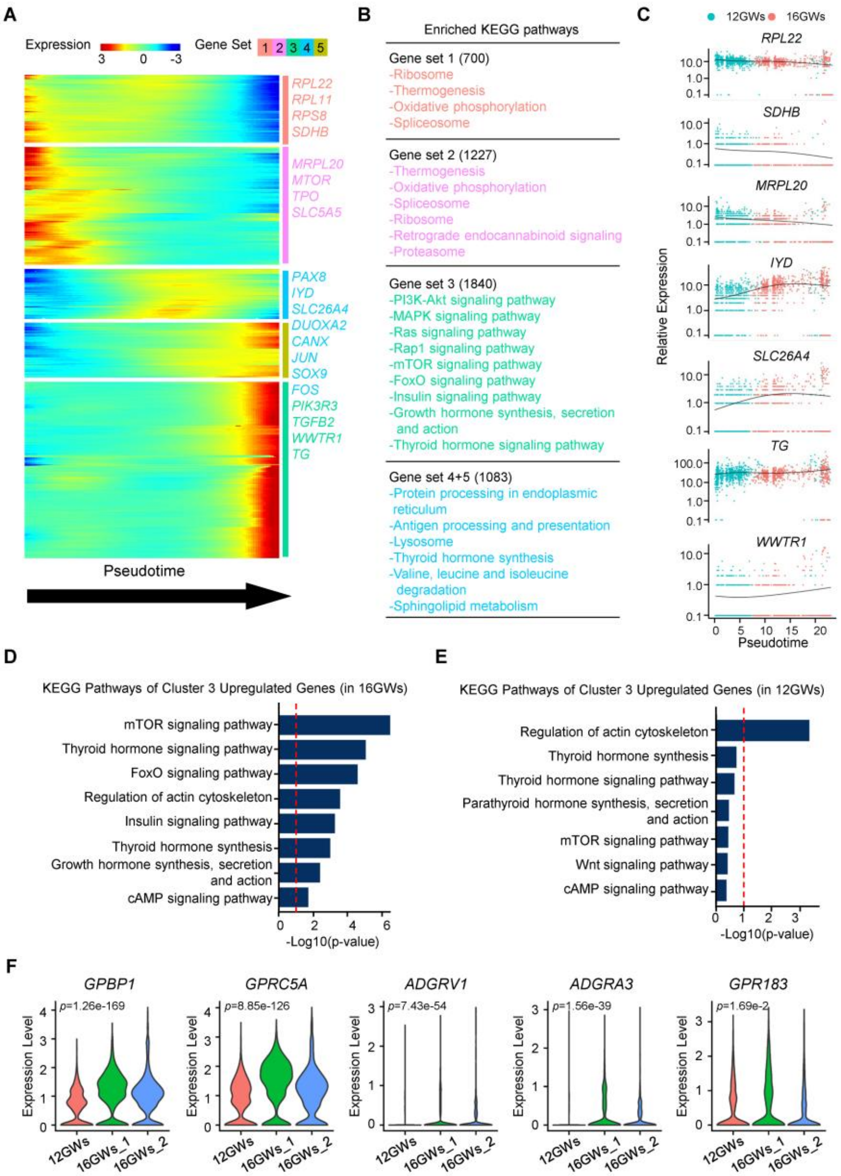

Figure S3. Pseudotime ordering, KEGG pathways and expression levels of differentially expressed genes. Related to Figure 2.

(A) Heatmap showing expression dynamics of differentially expressed genes ordered by pseudotemporal expression pattern. Pseudotemporal ordering is from left to right. Representative genes are shown for each gene set along the differentiation trajectory. (B) Enriched KEGG pathways of differentially expressed genes in each gene set. (C) Expression of RPL22, SDHB, MRPL20, IYD, SLC26A4, TG and WWTR1 along the pseudotime trajectory. (D-E) KEGG analysis of cluster 3 differentially expressed genes at 16GWs and 12GWs respectively. Differentially expressed genes were obtained by Comparing cells' transcriptome of cluster3 and other clusters (cluster1 and cluster2) in 16GWs or in 12GWs. (F) Violin plot visualizing expression levels of indicated genes in the three independent samples. p-value was performed by Wilcoxon Rank Sum test between 16GWs against 12Gws.

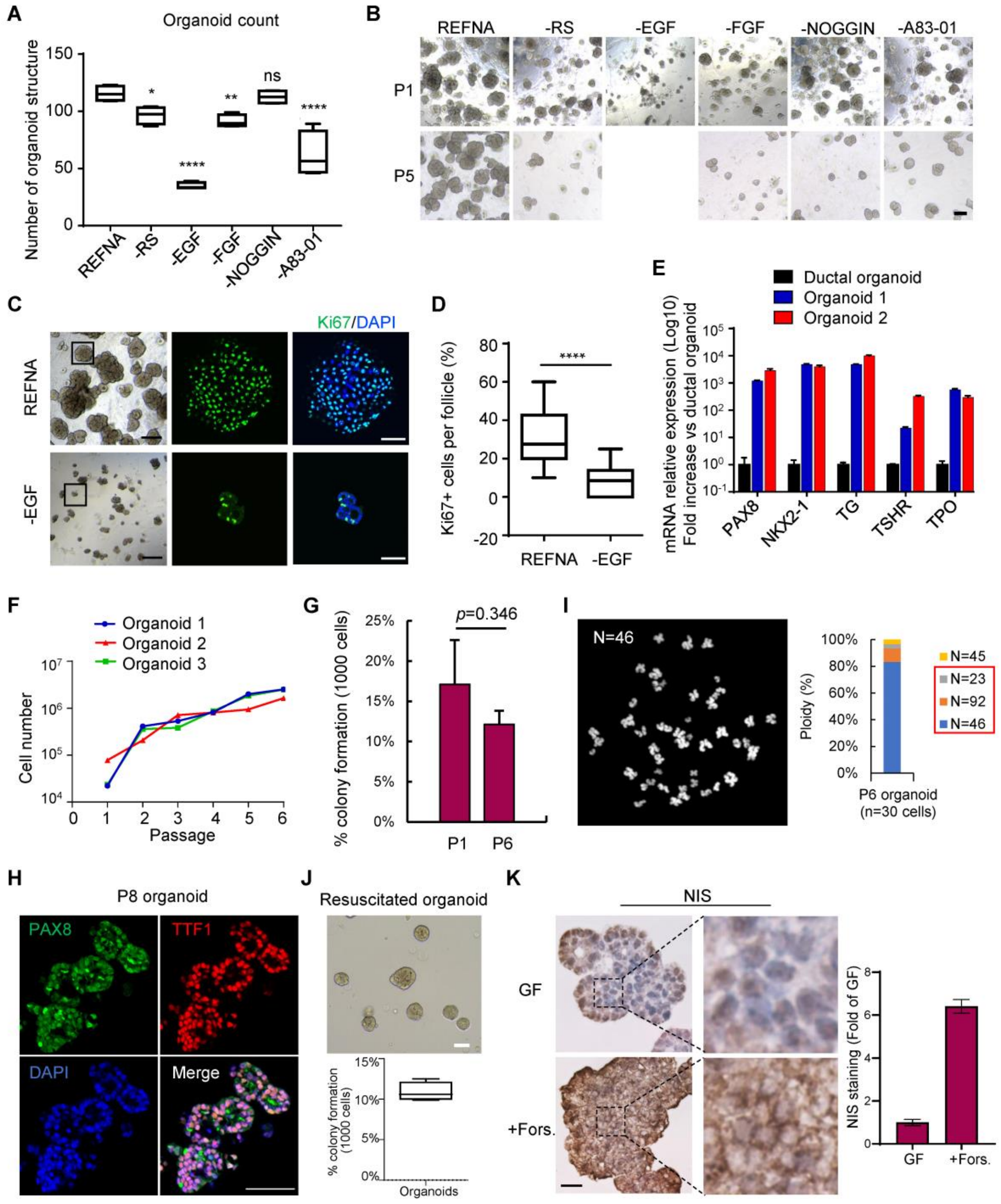

Figure S4. Verifications of optimal culture medium and characteristics of the fetal thyroid organoids. Related to Figure 3.

(A) Effect of organoid structure number of withdrawing growth factors from expansion medium. A total of 4 independent experiments were analyzed. Adjusted p_value (one-way ANOVA) * $p < 0.05$; ** $p < 0.01$; **** $p < 0.0001$; ns, not significant. (B) Representative brightfield images of organoids that were cultured in elimination of indicated factor conditions (scale bar=100 μm). P1=passage 1, P5=passage 5. (C) on the left panel: Representative brightfield images of organoids in culturing

conditions REFNA and –EGF (scale bar=100 μm); on the right panels: Immunofluorescence of ki67 of organoids in culturing conditions REFNA and –EGF (scale bar=50 μm). (D) Percentage of ki67+ cells per follicle in culturing conditions of (C). N=20; data are represented as mean ± SD; **** $p < 0.0001$. (E) qRT-PCR analysis of thyrocyte marker gene expression in thyroid organoids from two independent fetuses. The negative control was an unrelated liver ductal organoid. The dataset of the human protein atlas (https://www.proteinatlas.org/) shows that thyrocyte maker genes are undetectable in liver tissue. (F) Growth curve of fetal thyroid organoids from three independent fetuses followed from Passage 1 to Passage 6. The organoids were passaged every 10-14 days. (G) Percentage of colony formation per 1,000 thyrocytes from fetal thyroid organoids in Passage 1 and Passage 6. Experiments were performed in two independent hFTOs. Data are represented as mean ± SD. (H) The double-labelling immunofluorescence of PAX8 and TTF1 of P8 hFTOs, scale bar: 100μm. P8=passage 8. (I) Karyotyping of organoid cells in the 6th passage. n = 30 cells. (J) Representative image of resuscitated fetal thyroid organoids (scale bar=100 μm). And the percentage of colony formation per 1,000 thyrocytes from resuscitated organoids. Data are represented as mean ± SD. (K) The immunohistochemistry of NIS (also known as SLC5A5) of the fetal thyroid organoids. GF indicated organoids cultured in growth-factor medium; +Fors. indicated organoids cultured in forskolin treatment condition. The enlarged view showing NIS is detectable in cell membrane of +Fors. organoids. The nuclei were stained with hematoxylin. Scale bar=20μm.

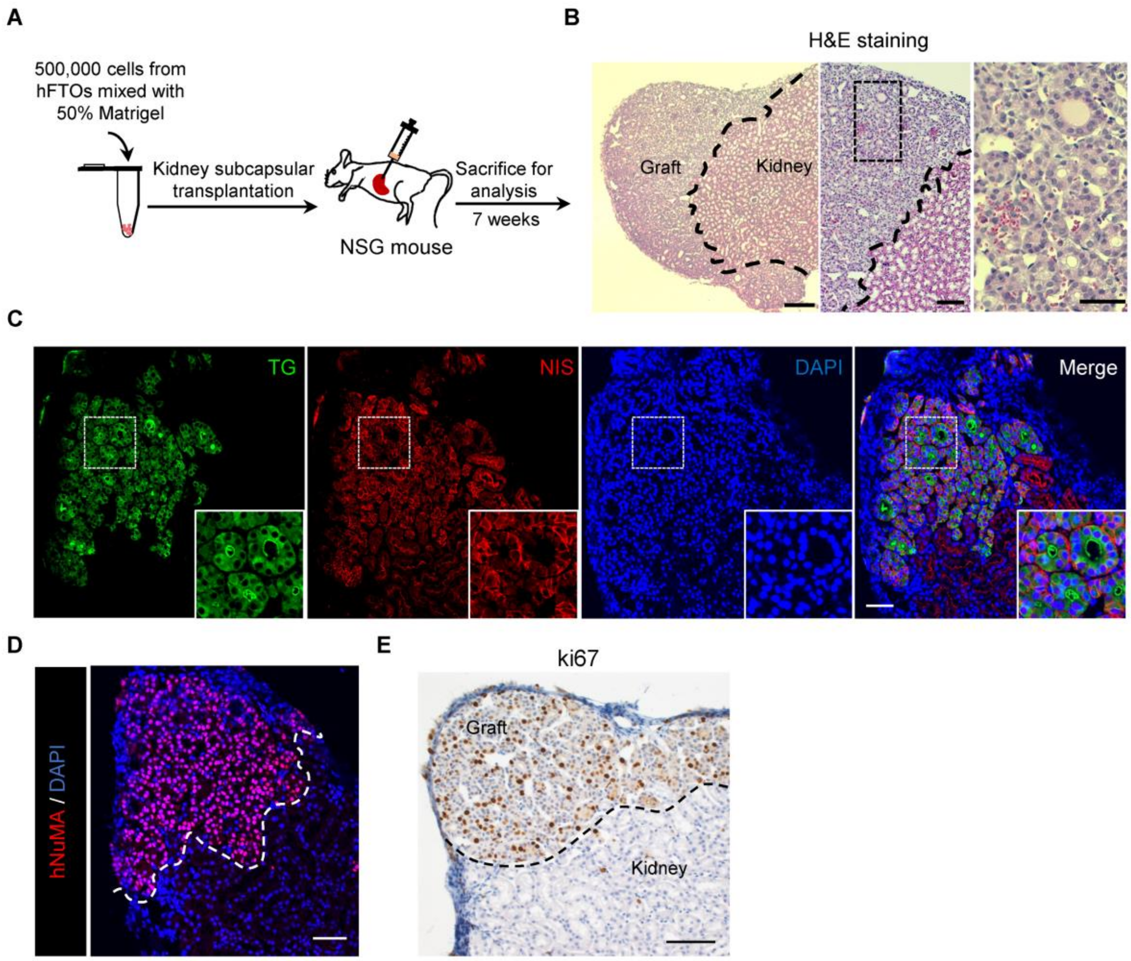

Figure S5. hFTOs display thyroid follicle generating ability *in vivo*. Related to Figure 3.

(A) Schematic design for fetal-derived thyroid organoids transplantation under the kidney capsule of NSG mouse. (B) Hematoxylin and eosin (H&E) staining on grafted kidney section showed the localization of the graft in the cortical area of the host organ (left panel) and monolayer cuboidal epithelium organization of follicles (right panels). Black dashed line divided the graft and kidney tissue. Scale bars from left to right: 200μm, 100μm and 50μm. (C) The double-labelling immunofluorescence of TG and NIS in the graft tissue, scale bar: 50μm. (D) The immunofluorescence of hNuMA (a human marker for nucleus) and DAPI in the graft tissue. White dashed line divided the graft and kidney tissue. Scale bar: 50μm. (E) The immunohistochemistry of Ki67 in the graft tissue. Black dashed line divided the graft and kidney tissue. Scale bar=200μm.

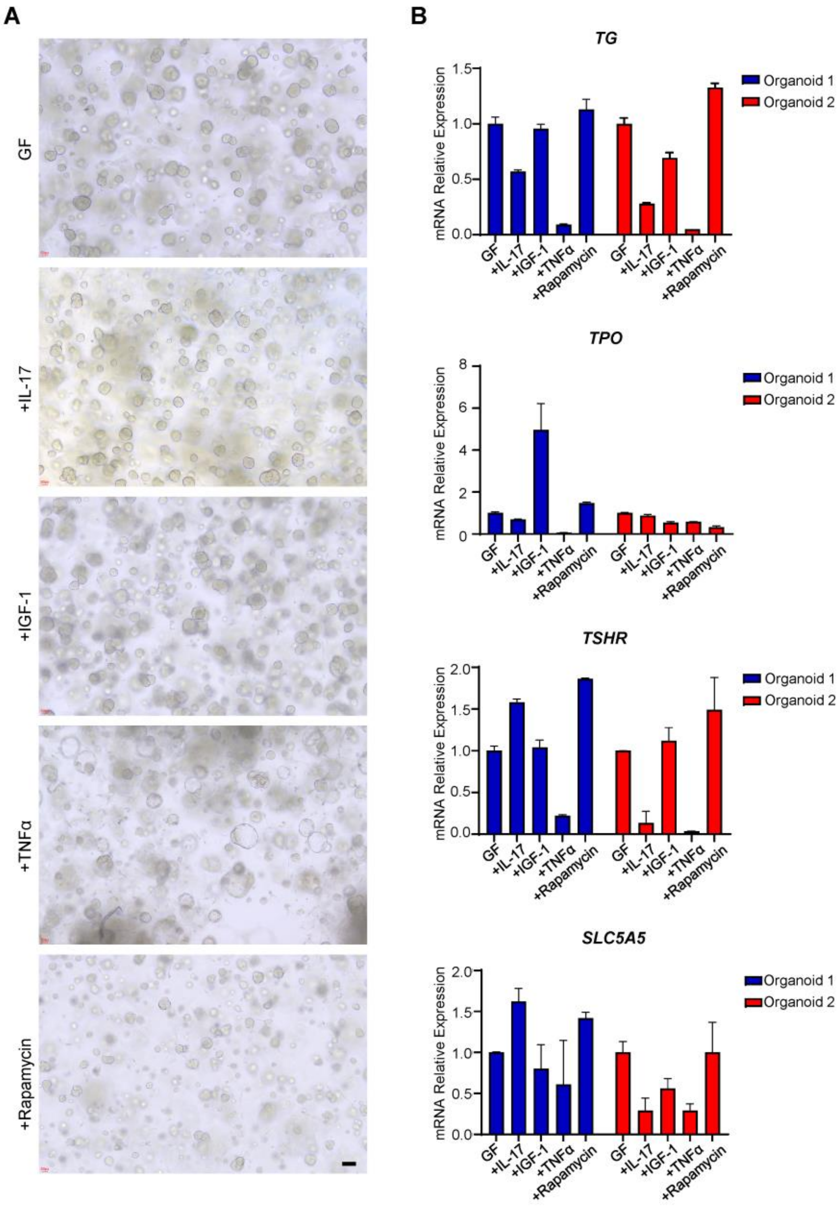

Figure S6. Modulate different signaling pathways in fetal thyroid organoid. Related to Figure 4. (A) Representative images of thyroid organoids modulated by different signals for 14 days. (B) qRT-PCR analysis of thyrocyte markers gene expression of organoids from (A). Two replicates (organoid 1 and organoid 2) from different donors were used. (Notes: GF stands for growth factor medium; +IL-17 refers to the treatment condition of 50 ng/ml IL-17; +IGF-1 is the condition of 100 ng/ml IGF-1; +TNFa stands for condition of 50 ng/ml TNFa; +Rapamycin indicated the condition of 50 nM rapamycin. Scale bar=100μm).

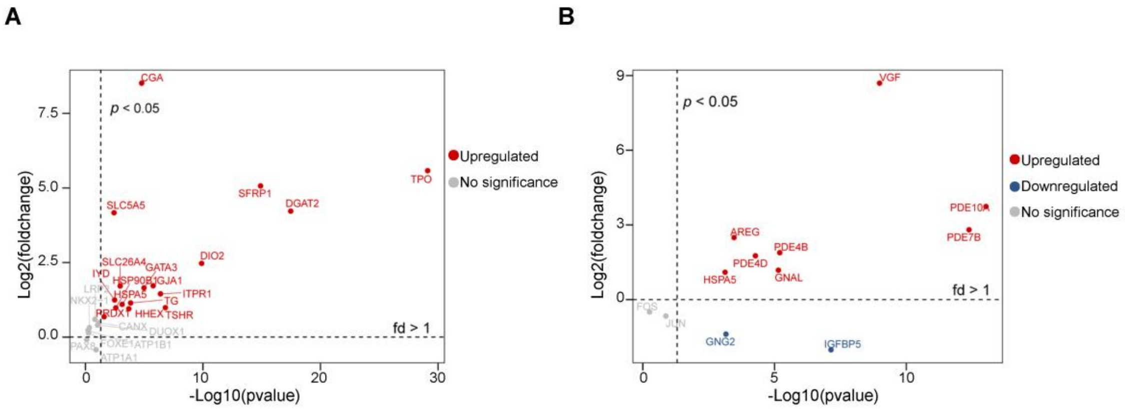

Figure S7. Differential expression analysis of gene sets in Figure 5C (A) and Figure 5D (B) by DEseq2. Related to Figure 5. The genes significantly upregulated or downregulated in hMTOs ($p<0.05$) are marked in red or blue. The grey dots indicate that gene has no significant differential expression between hFTOs and hMTOs.

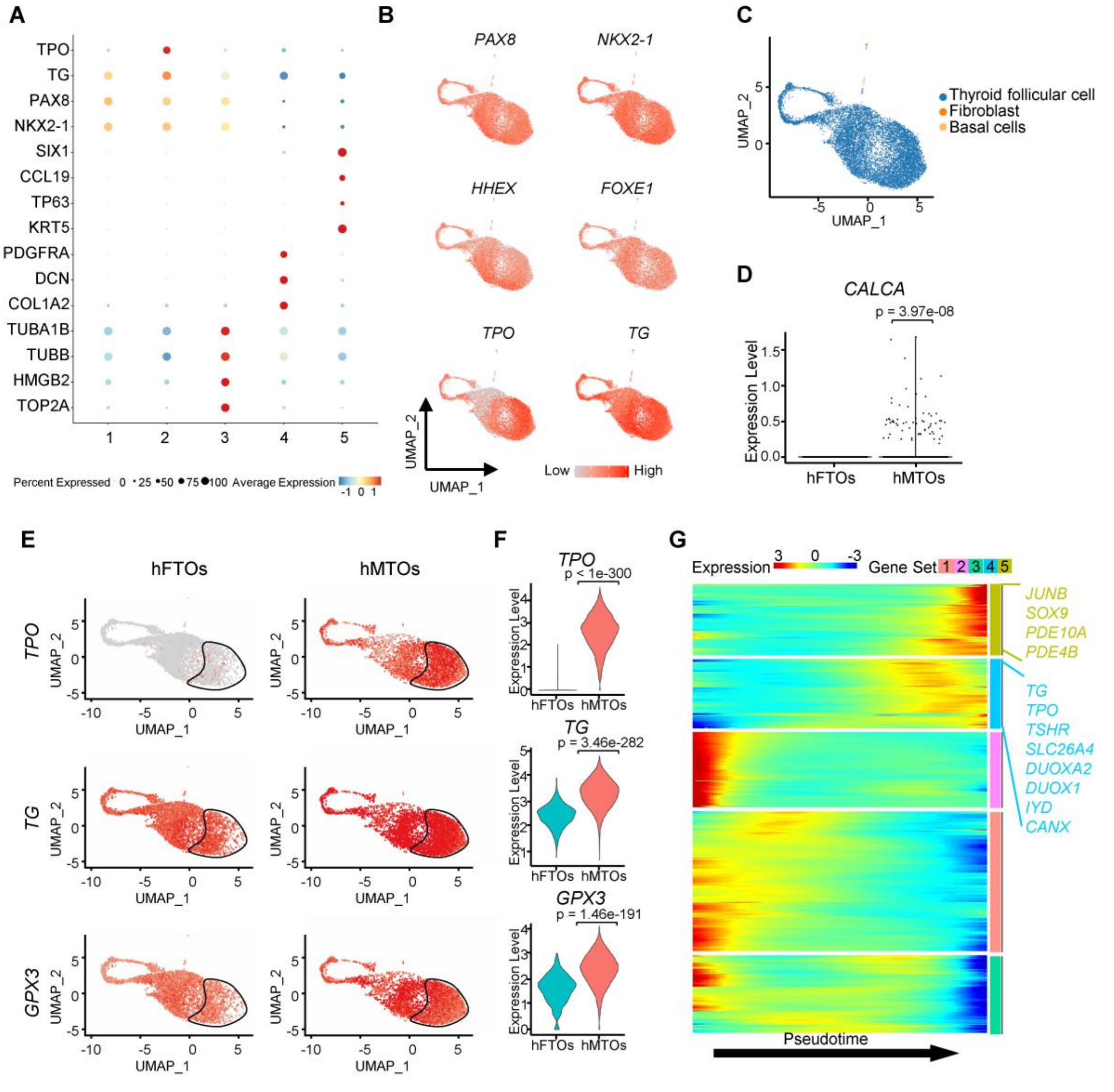

Figure S8. scRNA-Seq analysis of thyroid organoids reveals heterogeneity. Related to Figure 6. (A) Dot plot of known markers for distinct cell types, with color representing the average expression within cluster and dot size representing the percentage of cells within cluster expressing the indicated gene. (B) UMAP plot showing the expression levels of known transcriptional factors and thyroid marker genes. (C) UMAP plot of single-cell transcriptomes from thyroid organoids, colored and annotated by distinct cell types. (D) Expression of CALCA in single-cell sequencing data of hFTOs and hMTOs. Each dot represents one cell. p-value was calculated by the Wilcoxon Rank Sum test. (E) UMAP plot for expression levels of indicated genes in hFTOs and hMTOs. A gradient of orange color indicates low to high expression levels. (F) Violin plot visualizing expression levels of indicated genes in hFTOs and hMTOs. Color coded hFTOs (blue) and hMTOs (red). p-value was calculated by the Wilcoxon Rank Sum test. (G) Expression of differentially expressed genes ordered by pseudotime. Pseudotemporal ordering is from left to right. Selected representative genes are shown for each gene set along the differentiation trajectory.

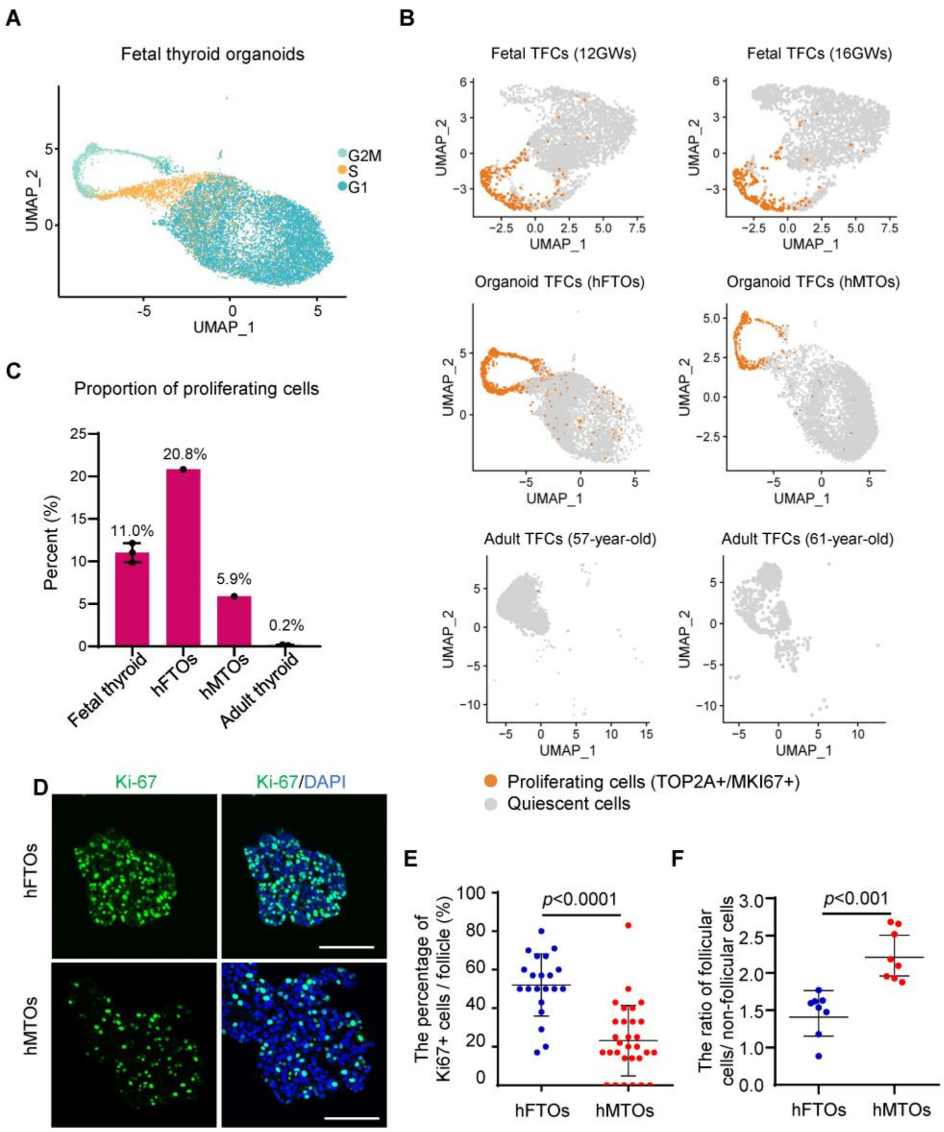

Figure S9. The proportion of proliferation cells in thyroid organoids and thyroid tissue. Related to Figure 6.

(A) UMAP plot showing cell cycle stages of cells in fetal thyroid organoids. (B) UMAP plot showing TOP2A+/MKI67+ cells of TFCs in fetal thyroid, thyroid organoids (hFTOs and hMTOs) and adult thyroid, respectively. Positive for TOP2A/MKI67 indicates the proliferation status of cells while the negative cells we defined as quiescent status. (C) Analysis of single-cell sequencing data showing percentage of proliferating TFCs of fetal thyroid (n=3), hFTOs (n=1), hMTOs (n=1) and adult thyroid (n=2), respectively. (D) Representative Ki67 and DAPI immunofluorescent staining images of hFTOs and hMTOs. Scale bar=100μm. (E) Quantification of the percentage of ki67+ cells per follicle from (D). Adjusted pvalue (Unpaired t test) $p < 0.0001$. n=21 for hFTOs and n=30 for hMTOs. (F) Quantification of the ratio of follicular cells to non-follicular cells. Adjusted pvalue (Unpaired t test) $p < 0.001$. n=8 for hFTOs and n=8 for hMTOs.

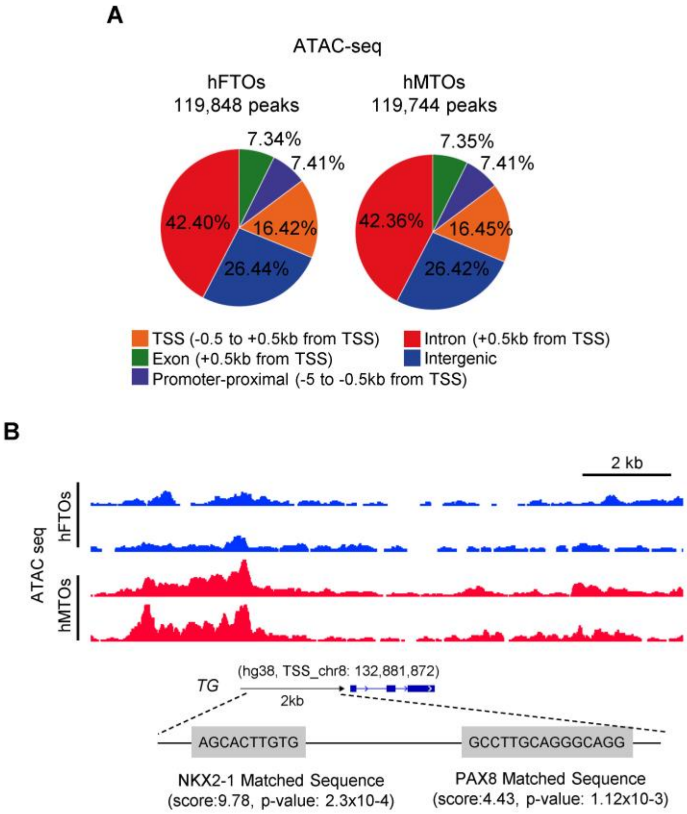

Figure S10. Chromatin accessibility determined the fate of human thyroid cells. Related to Figure 7.

(A) Distribution of chromatin accessible ATAC peaks on genome in hFTOs and hMTOs. (B) ATAC-seq signals at TG genome site of two biological replicates in hFTOs and hMTOs displayed by the integrative genomics viewer. Pax8 and NKX2.1 motif scanning at TG genome site using MEM Suite website. Scale bar=2 kilobase (kb).

Table S1. List of fetal and adult samples information.

Related Figures	Name	Gestation weeks (GWs)	Tissue	Donor age (years)	Diagnosis	Assay in this study
Fig. 1 and Fig. 2	12GWs	12	thyroid	-	healthy	scRNA-seq
	16GWs_1	16	thyroid		healthy	scRNA-seq
	16GWs_2	16	thyroid	-	healthy	scRNA-seq
Fig. 2	12GWs	12	thyroid	-	healthy	IHC staining
	16GWs	16	thyroid	-	healthy	IHC staining
Fig. 3 and Fig. 4	Organoid 1	12.5	thyroid	-	healthy	organoid culture
	Organoid 2	12	thyroid	-	healthy	organoid culture
	Organoid 3	12	thyroid	-	healthy	organoid culture
Fig. 4	GF/+Fors. 1	12	thyroid	-	healthy	organoid culture
	GF/+Fors. 2	12	thyroid	-	healthy	organoid culture
Fig. 5	hFTOs/hMTOs_1	12	thyroid	-	healthy	bulk RNA-seq
	hFTOs/hMTOs_2	12	thyroid	-	healthy	bulk RNA-seq
	hFTOs/hMTOs_3	12.5	thyroid	-	healthy	bulk RNA-seq
	Fetal_T1	12	thyroid	-	healthy	bulk RNA-seq
	Fetal_T2	12	thyroid	-	healthy	bulk RNA-seq
	Fetal_T3	12.5	thyroid	-	healthy	bulk RNA-seq
	Fetal_T4	12.5	thyroid	-	healthy	bulk RNA-seq
	Adult_T1	-	thyroid	31	N(PTC)	bulk RNA-seq
	Adult_T2	-	thyroid	53	N(PTC)	bulk RNA-seq
Fig. 6	hFTOs/hMTOs	12	thyroid	-	healthy	scRNA-seq
Fig. 7	hFTOs/hMTOs_1	12	thyroid	-	healthy	ATAC-seq
	hFTOs/hMTOs_2	12.5	thyroid	-	healthy	ATAC-seq

*N(PTC) - papillary thyroid carcinoma, morphologically normal thyroid tissue.

Table S2. List of antibodies source and primers of qRT-PCR.

Target	Antibody	company	catalog No.
FOXO1	Anti-FOXO1A antibody	abcam	ab52857
MAFB	MafB Antibody	R&D	MAB3810-SP
STAT3	Stat3 (124H6) Mouse mAb	CST	9139
ZO-1	ZO-1 Polyclonal Antibody	Proteintech	21773-1-AP
TG	Anti-Thyroglobulin	Proteintech	60272-1-Ig
SLC5A5	Anti-Sodium Iodide Symporter	Proteintech	24324-1-AP
PAX8	PAX8 Monoclonal antibody	Proteintech	60145-4-Ig
NKX2-1	Anti-TTF1 antibody	abcam	ab227652
T4	Anti-Thyroxine	MP	DS_0865850
Ki-67	Anti- Ki-67	BD	550609
NuMA	Anti-NuMA	Abcam	ab97585

Target	Primers 5'-3'
NKX2-1	Forward- GGACGGGAGCTGGGGAGAGG
NKX2-1	Reverse- ATTTTCGCGGAGGGCGGTCG
PAX8	Forward- CAACCTCCCTATGGACAGC
PAX8	Reverse- CATCCGTGCGAAGGTGCTT
TG	Forward- GGCTAATGCTACATGTCCTG
TG	Reverse- GCTTCTGTTGGAGATGCTGG
TPO	Forward- GTCTGTCACGCTGGTTATGG
TPO	Reverse- CAATCACTCCGCTTGTTGGC
TSHR	Forward- GAACTGATAGCAAGAAACACCTGG
TSHR	Reverse- GTATCCTGGAACTTGGACTTTT
SLC5A5	Forward- TGTCTGCCAGCTTCATGTCG
SLC5A5	Reverse- GGTGTACAGCATCGTGGCTA
GADPH	Forward- GACTCATGACCACAGTCCATGC
GADPH	Reverse- AGAGGCAGGGATGATGTTCTG

Table S3. List of KEGG pathways significantly enriched in cluster 3. (Related to Figure 1)

ID	Description	GeneRatio	BgRatio	pvalue	p.adjust	Count	symbol
hsa00310	Lysine degradation	8/154	63/8105	2.26E-05	0.005384	8	NSD1/KMT2E/KMT2A/KMT2C/PHYKPL/ASH1L/PLOD2/AASS
hsa04520	Adherens junction	8/154	71/8105	5.47E-05	0.006504	8	AFDN/ACTN4/ACTN4/BAIAP2/FER/CTNNB1/PTPRM/CDH1/TCF7
hsa04140	Autophagy - animal	10/154	141/8105	0.000336	0.017879	10	LAMP1/LAMP2/AKT2/RB1CC1/ITPR1/TP53INP2/RRAGB/TSC1/MAPK10/PIK3R3
hsa05017	Spinocerebellar ataxia	10/154	143/8105	0.000376	0.017879	10	AKT2/ATXN2/RB1CC1/ATP2A2/ITPR1/ATXN1/RORA/PRKCA/MAPK10/PIK3R3
hsa04919	Thyroid hormone signaling pathway	9/154	121/8105	0.000471	0.017879	9	MED13/AKT2/MED13L/ATP2A2/MED17/CTNNB1/PLCE1/PRKCA/PIK3R3
hsa05205	Proteoglycans in cancer	12/154	205/8105	0.00051	0.017879	12	PPP1CB/AKT2/SMAD2/PTK2/ANK3/ITPR1/BRAF/CTNNB1/PLCE1/PRKCA/PIK3R3/STAT3
hsa05165	Human papillomavirus infection	16/154	331/8105	0.000528	0.017879	16	AKT2/ATP6V1H/DLG1/PTK2/IRF1/LAMA5/COL4A1/PATJ/MAML2/MAGI1/MAGI1/ATP6V0A1/CTNNB1/TSC1/PIK3R3/CSNK1A1/TCF7
hsa04933	AGE-RAGE signaling pathway in diabetic complications	8/154	100/8105	0.000601	0.017879	8	AKT2/SMAD2/COL4A1/PLCE1/PRKCA/MAPK10/PIK3R3/STAT3
hsa04150	mTOR signaling pathway	10/154	155/8105	0.000711	0.0182	10	LPIN2/AKT2/ATP6V1H/CLIP1/FNIP1/BRAF/RRAGB/TSC1/PRKCA/PIK3R3
hsa05213	Endometrial cancer	6/154	58/8105	0.000765	0.0182	6	AKT2/BRAF/CTNNB1/PIK3R3/CDH1/TCF7
hsa04931	Insulin resistance	8/154	108/8105	0.001001	0.021649	8	PPP1CB/AKT2/MLXIP/MLXIP/OGT/PRKAG2/MAPK10/PIK3R3/STAT3

hsa04910	Insulin signaling pathway	9/154	137/8105	0.001155	0.021859	9	PPP1CB/AKT2/PHKB/EXOC7/BRAF/TSC1/PRKAG2/MAPK10/PIK3R3
hsa05210	Colorectal cancer	7/154	86/8105	0.001194	0.021859	7	AKT2/SMAD2/BRAF/CTNNB1/MAPK10/PIK3R3/TCF7
hsa05225	Hepatocellular carcinoma	10/154	168/8105	0.00132	0.02244	10	AKT2/SMAD2/BRAF/ARID2/CTNNB1/ARID1B/PRKCA/PIK3R3/CSNK1A1/TCF7
hsa04510	Focal adhesion	11/154	201/8105	0.001511	0.023977	11	ACTN4/ACTN4/PPP1CB/AKT2/PTK2/BRAF/LAMA5/COL4A1/CTNNB1/PRKCA/MAPK10/PIK3R3
hsa04935	Growth hormone synthesis, secretion and action	8/154	119/8105	0.001871	0.027826	8	AKT2/PTK2/ITPR1/ADCY9/PRKCA/MAPK10/PIK3R3/STAT3
hsa05223	Non-small cell lung cancer	6/154	72/8105	0.002374	0.033242	6	AKT2/BRAF/FHIT/PRKCA/PIK3R3/STAT3
hsa04918	Thyroid hormone synthesis	6/154	75/8105	0.002921	0.03716	6	DUOX1/ITPR1/ADCY9/PRKCA/HSPA5/IYD
hsa04024	cAMP signaling pathway	11/154	219/8105	0.002968	0.03716	11	AFDN/PPP1CB/AKT2/ATP2A2/BRAF/PLCE1/RYR2/ADCY9/MAPK10/PIK3R3/PDE4C
hsa05212	Pancreatic cancer	6/154	76/8105	0.003123	0.03716	6	AKT2/SMAD2/BRAF/MAPK10/PIK3R3/STAT3
hsa05163	Human cytomegalovirus infection	11/154	225/8105	0.003651	0.041379	11	AKT2/PTK2/AKAP13/ITPR1/CTNNB1/TSC1/ADCY9/PRKCA/PIK3R3/STAT3/PDIA3
